# Limitations on Temporal Processing by Cochlear Implant Users: A Compilation of Viewpoints

**DOI:** 10.1177/23312165251317006

**Published:** 2025-03-17

**Authors:** Robert P. Carlyon, John M. Deeks, Bertrand Delgutte, Yoojin Chung, Maike Vollmer, Frank W. Ohl, Andrej Kral, Jochen Tillein, Ruth Y. Litovsky, Jan Schnupp, Nicole Rosskothen-Kuhl, Raymond L. Goldsworthy

**Affiliations:** 1 Cambridge Hearing Group, MRC Cognition & Brain Sciences Unit, 2152 University of Cambridge, Cambridge, UK; 2 465573 Eaton-Peabody Laboratories, Massachusetts Eye and Ear, Boston, MA, USA; 3 Department of Experimental Audiology, University Clinic of Otolaryngology, Head and Neck Surgery, 493262 Otto von Guericke University Magdeburg, Magdeburg, Germany; 4 528040 Leibniz Institute for Neurobiology (LIN), Magdeburg, Germany; 5 Institute of Audio-Neuro-Technology & Department of Experimental Otology, Clinics of Otolaryngology, Head and Neck Surgery, Hannover Medical School, Hannover, Germany; 6 Clinics of Otolaryngology, Head and Neck Surgery, J.W.Goethe University, Frankfurt, Germany; 7 MedEl Company, Hannover, Germany; 8 49501 Waisman Center, University of Wisconsin-Madison, Madison, WI, USA; 9 Gerald Choa Neuroscience Institute and Department of Otolaryngology, Chinese University of Hong Kong, Hong Kong (NB Hong Kong is a Special Administrative Region) of China; 10 Neurobiological Research Laboratory, Section for Experimental and Clinical Otology, Department of Oto-Rhino-Laryngology, 14879 Medical Center, Faculty of Medicine, University of Freiburg, Freiburg, Germany; 11 505887 Bernstein Center Freiburg & Faculty of Biology, University of Freiburg, Freiburg, Germany; 12 Auditory Research Center, Caruso Department of Otolaryngology, 12223 Keck School of Medicine, University of Southern California, Los Angeles, CA, USA

**Keywords:** cochlear implant, plasticity, pitch perception/ localization, inter-aural time difference

## Abstract

Cochlear implant (CI) users are usually poor at using timing information to detect changes in either pitch or sound location. This deficit occurs even for listeners with good speech perception and even when the speech processor is bypassed to present simple, idealized stimuli to one or more electrodes. The present article presents seven expert opinion pieces on the likely neural bases for these limitations, the extent to which they are modifiable by sensory experience and training, and the most promising ways to overcome them in future. The article combines insights from physiology and psychophysics in cochlear-implanted humans and animals, highlights areas of agreement and controversy, and proposes new experiments that could resolve areas of disagreement.

## Introduction

Cochlear implants (CIs) have proven remarkably successful at restoring speech perception to severely and profoundly deaf people, at least in quiet situations. Under such conditions the more successful listeners achieve good open-set speech perception, despite the rather coarse representation of the speech signal provided by their device(s). Most contemporary CI processing strategies extract the envelope in each frequency band and use it to amplitude-modulate a fixed-rate pulse train applied to the corresponding electrode of the implant, thereby discarding the temporal fine structure (TFS) in the waveform. Combined with the spread of current along the cochlea and across the auditory nerve (AN) array, this means that the brain must extract speech from a slowly varying and rather blurred neural excitation pattern. Hence, in addition to the substantial clinical benefits, the remarkable success of many CI listeners in this task informs our understanding of how the brain does, or at least can, process speech (cf. Shannon et al., 1995).

**Figure 1.**
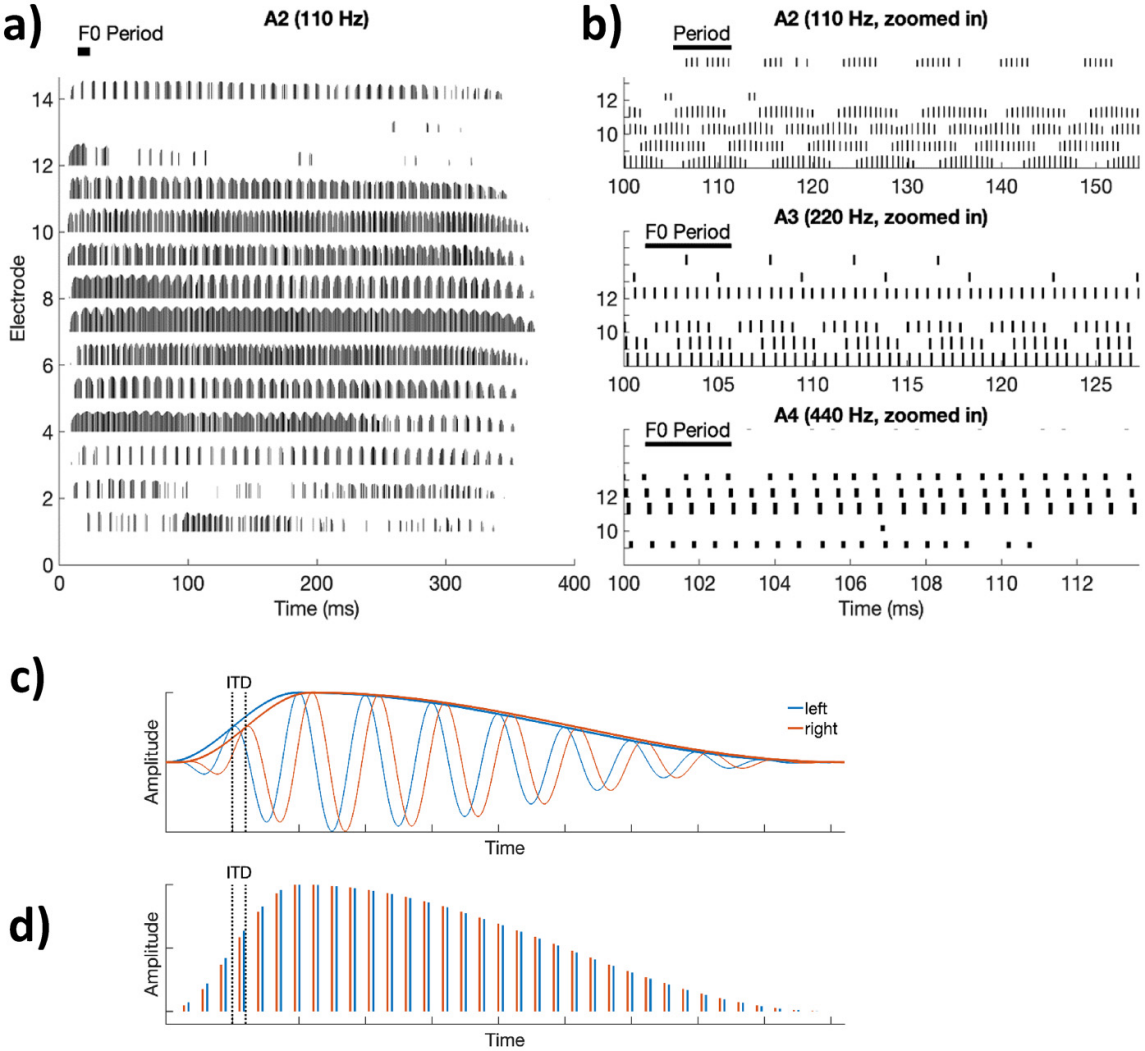
(a) Electrodogram showing the output of the ACE processing strategy to a piano note having an F0 of 110 Hz. The period of the waveform, equal to 9.1 ms, is shown by the solid horizontal line. (b) Zoomed-in versions of the outputs of channels 8–15 for notes with F0 s of 110, 220, and 440 Hz. (c) Schematic of the output of one analysis filter of CIs in the left (blue) and right (red) ear of a bilaterally implanted listener and where each output consists of a single amplitude-modulated sinusoid. Note that the fine structure and envelope both lead on the left ear relative to the right ear. (d) Pulse trains in the two ears resulting from the filtered waveforms in part (c). Note that because the outputs of the two CIs are not synchronized, the fine structure can lead on the right ear, as illustrated here, even though the direction of the ITD envelope is conveyed correctly. ACE = Advanced Combination Encoder; CI = cochlear implant; ITD = interaural time difference.

Unfortunately, even when CI listeners show good speech perception in quiet, they usually perform poorly on two tasks that depend on the processing of fine timing information. One task involves the use of temporal information to perceive pitch, which is important for the perception of melody, prosody, and for the comprehension of tonal languages. A second task concerns the use of interaural time differences (ITDs) to localize sounds, which is a major problem for bilaterally implanted listeners. Figure 1 illustrates how the removal of TFS cues by CI processors may impair pitch and ITD perception. Figure 1a shows the output of the Advanced Combination Encoder (ACE) processing strategy (Vandali et al., 2000) to a piano note having a fundamental frequency (F0) of 110 Hz. It can be seen that the F0 is encoded by the envelope in several frequency bands but that the envelopes are often quite shallow and not aligned across channels. The shallow modulation results from reverberation due to room acoustics and from the limited number of harmonics falling within each analysis band. This latter factor is illustrated in Figure 1b, which plots the output of a subset of channel to notes with F0 s of 110, 220, and 440 Hz, and shows the reduced modulation depth with increasing F0; in addition, CI processors usually low-pass filter the envelope in each channel, thereby further contributing to the reduction in modulation depth with increasing F0.

Figure 1c illustrates the bandpass filtered waveform of one channel of a CI that passes only one frequency component of the input, together with the same plot for the contralateral CI of a bilaterally implanted listener; the resulting pulse trains are shown in Figure 1d. Although the envelope ITD is represented in the pulse train, the carrier pulse trains in the two ears are unsynchronized with their own arbitrary ITD. Furthermore, tiny differences between the clock rates of the two processors can cause this irrelevant carrier ITD to vary over time (not shown), confounding the CI patient's spatial perception.

**Figure 2.**
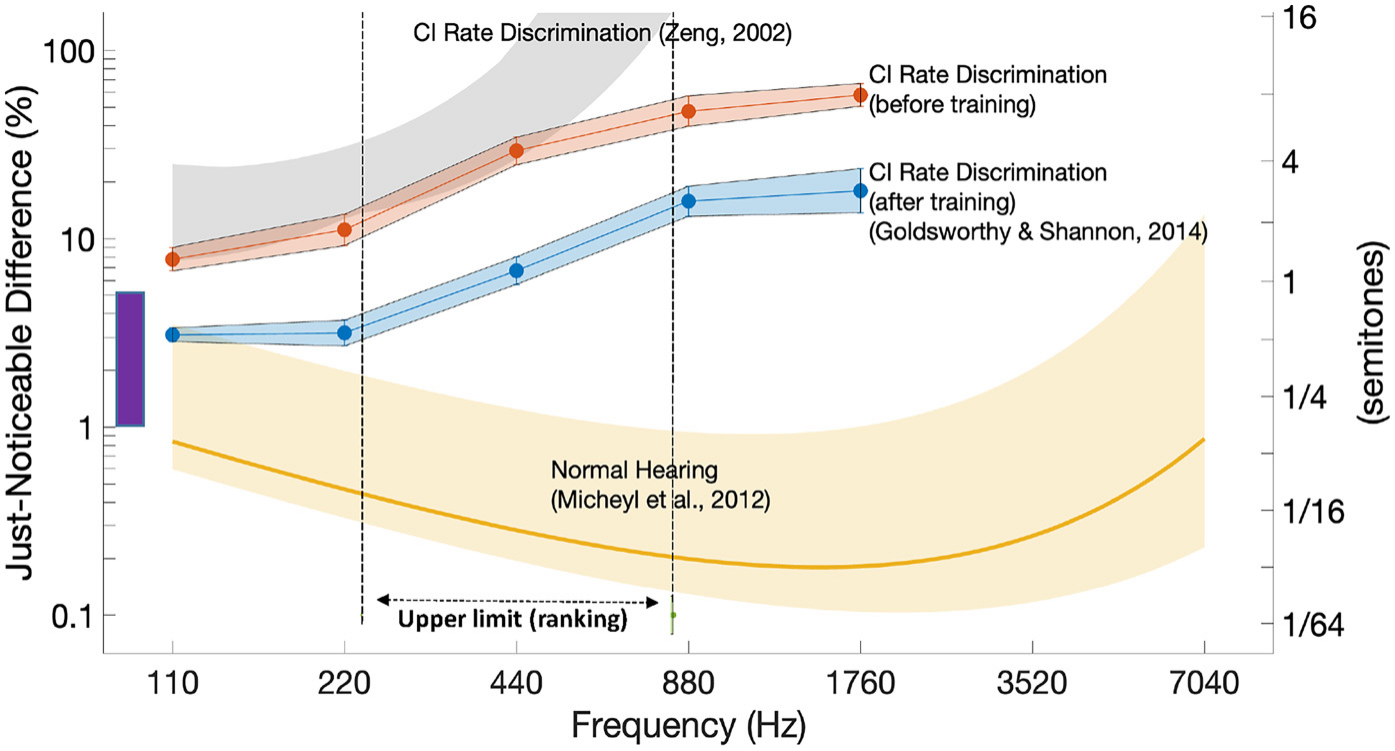
Rate difference limens (DLs) for pulse trains presented to one CI electrode are shown before and after extensive training by the red and blue symbols, respectively. The yellow line shows pure-tone DLs in NH. The dashed line with arrows at the bottom of the plot shows the range of “upper limits” for rate discrimination in the study by Carlyon et al. (2019). The purple bar shows the range of DLs for rate discrimination of bandpass-filtered pulse trains observed in a range of NH studies. CI = cochlear implant; NH = normally hearing.

The effects of contemporary CI processors on the cues necessary for pitch and ITD perception have prompted some companies to develop alternative strategies that preserve TFS on a subset of apical (low-frequency) channels (Büchner et al., 2008; Dhanasingh & Hochmair, 2021). Unfortunately, deficits in the temporal coding of pitch and ITDs by CI listeners persist even in experimental settings where the speech processor is bypassed and highly simplified stimuli are presented to one electrode (for pitch perception) or pair of electrodes (for ITD processing). These limitations have been the subject of experimental investigation for several decades (Shannon, 1983; Townshend et al., 1987; van Hoesel & Clark, 1997) and are described in detail in the following sections. Figure 2 compares pulse-rate discrimination thresholds as a function of the rate of a pulse train applied to a single CI electrode to pure-tone frequency discrimination thresholds for normally hearing (NH) listeners. The CI thresholds are not only much higher than the NH pure-tone thresholds, but also increase steeply with increasing pulse rate. Indeed, pitch-ranking studies reveal that, for most CI listeners, pitch does not increase with increases in pulse rate above some value, which is typically about 300 Hz but that varies across listeners, reaching 800–900 Hz in a small number of cases (dashed line with arrows at the bottom of Figure 2a; see also e.g., Kong & Carlyon, 2010). This “upper limit” raises the possibility that the CI rate-discrimination thresholds shown in Figure 2 at very high rates may be based on a percept other than pitch, and this point will be discussed in the next two sections of the present article.

**Figure 3.**
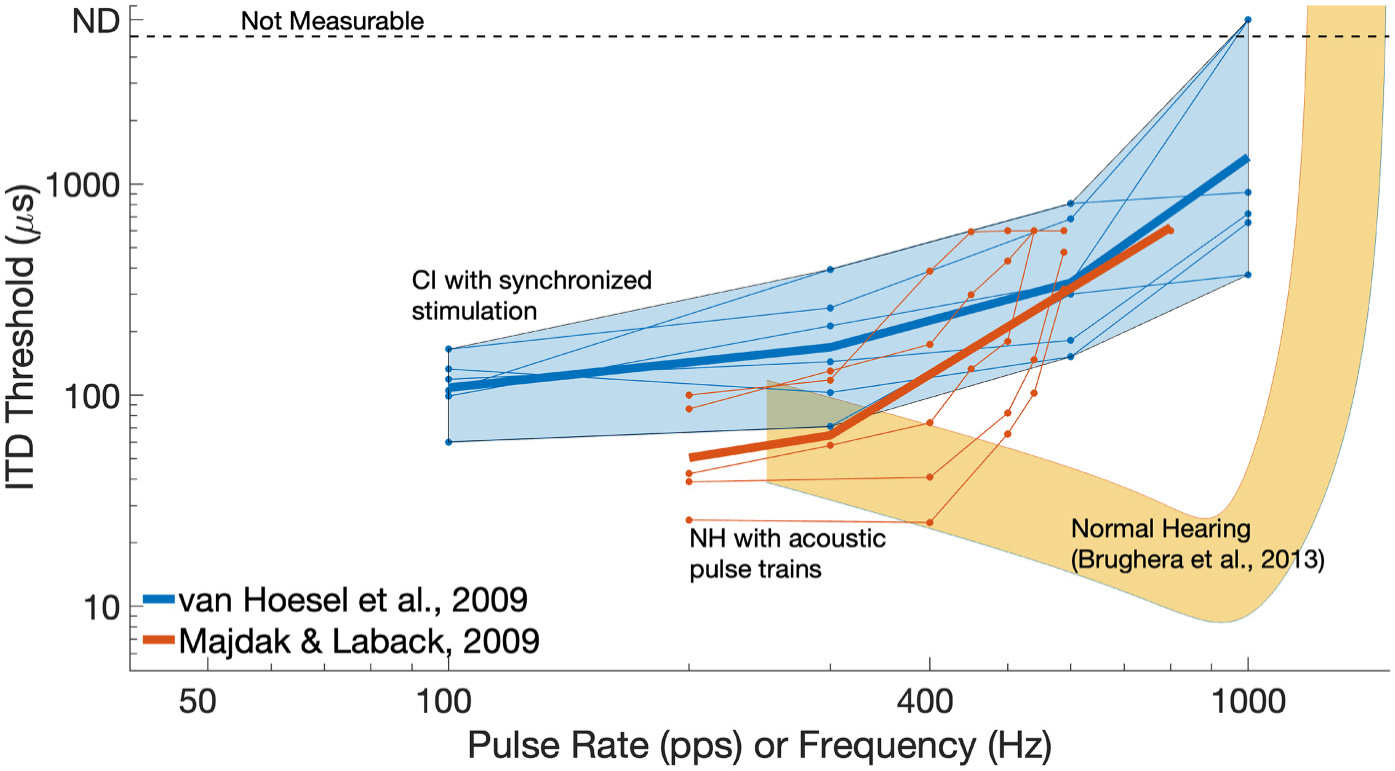
Blue lines: ITD thresholds as a function of pulse rate from a study by van Hoesel et al. (2009) that bypassed the speech processor and presented simple pulse trains to CI listeners. Red lines: ITD thresholds as a function of the rate of bandpass-filtered acoustic pulse trains presented to NH listeners (van Hoesel et al, 2009). Faint lines show data from individual participants; solid-lines show broken-stick fits to the mean data from each study. Yellow shaded area: data obtained from pure tones presented to NH listeners (Brughera et al., 2013).

ITD processing by bilaterally implanted listeners is also worse than in NH listeners, again even for idealized stimuli, and deteriorates with increases in pulse rate, as illustrated in Figure 3 for data reported by van Hoesel et al. (2009), and summarized for a wide range of studies in the review by Laback et al. (2015). Also similar to monaural rate discrimination, ITD discrimination varies markedly across listeners, both in terms of the overall size of thresholds and of the upper limit. For example, although ITD discrimination thresholds typically increase markedly or become unmeasurable for rates above about 300 pulses per second (pps; Figure 3), some exceptional CI users are sensitive to ongoing ITDs at considerably higher rates (e.g., 600 pps for two listeners in van Hoesel et al., 2009 and one of the four listeners in Laback et al., 2007).

The data summarized in Figures 2 and 3 raise an interesting conundrum that, in a sense, is opposite to the issue of how CI listeners can achieve good speech perception from a highly degraded peripheral representation: Why is the sensitivity of CI listeners to changes in pulse rate and in ITD so poor, even when clear and unambiguous information is presented? To address this issue, each section of the present article provides an opinion piece from seven research groups with expertise in the temporal processing of CI stimulation. Each section provides a different perspective on the problem, focusing primarily either on physiological data from animals or on human psychophysics, and on either pitch or ITD processing. However, although the perspectives differ, each contributor was asked to address the same set of questions. This approach was inspired by the multi-author “lack of consensus” article by Verschooten et al. (2019) on the controversy surrounding the highest frequency at which phase-locking is important for pitch perception in NH. It differs from that article not only in addressing monaural and binaural temporal processing in response to electrical stimulation by CI listeners rather than to acoustic stimulation in NH, but also in focusing on the roots of the limitations in temporal processing both at low and at high stimulation rates. The result here was a combination of converging evidence from different disciplines and authors on some issues and disagreement on others.

The first question posed to all contributors was: *To what extent are the limits on CI users’ use of purely temporal cues to perceive the pitch and spatial location of sounds (a) due to a fundamental biological limitation, and (b) modified by the presence and type of electrical stimulation that they have experienced?* Answers to part (a) addressed questions such as the neural stage(s) at which limitations in TFS processing likely arise, and whether the limitations are specific to electrical stimulation *per se*. Part (b) addressed the important issue of plasticity and of whether the removal of TFS by speech processors has limited the processing of fine timing cues by CI listeners, even when those processors are bypassed. The answers to both parts of the questions are intertwined, because the neural locus of the limitations may inform the likelihood of their modification by experience, and because the presence of neural plasticity may constrain the likely neural locus. In practical, clinical terms, the question could be rephrased as two thought experiments. First, if we provided newly implanted congenitally deaf infants with processors that accurately conveyed pitch and ITD TFS cues, how good would their perception of those cues be in adulthood, compared to people who had only ever been fitted with conventional processors, when the processor is removed and idealized stimuli presented to the CI electrodes? Second, in adult CI listeners, to what extent could sensitivity to these cues, assessed using idealized stimuli, be restored either by continued exposure to TFS-preserving processors or by extensive training? As with the early research on speech perception, the answers to these questions not only have clear and important clinical implications, but also provide insights into auditory processing and to sensory plasticity in general. We next invited authors to answer the question: *What would change your mind?*. We expected some controversy concerning the role of plasticity in particular, and so considered it important, to quote Verschooten et al. (2019), “to put the authors on the spot: for them to demonstrate that their theoretical position is falsifiable (and hence is science rather than dogma), and to commit them to changing their mind, should the results turn against them.”

The last question asked of each author was: *What if anything can be done to improve the temporal processing of pitch and localization cues by CI listeners?* It is related to our question about plasticity to the extent that improvements can be achieved by early exposure to, or training with, TFS cues, but extends the debate to other methods, such as changes to speech-processing strategies or to the method of stimulating the electrode array. Here the authors bring their scientific knowledge to explore the reasons for the limited success of existing attempts to improve pitch and ITD processing, and to propose modifications or replacements to those attempts.

Our final section summarizes the areas of agreement and highlights the areas of controversy. To aid the reader we provide a short bullet-point summary of each contributor's main arguments, focusing on the issues where there is most disagreement, namely the role of auditory experience and the potential for overcoming these temporal limitations. We hope that both the consensus and controversy summarized here will prove informative both to academic research groups and to CI companies in their efforts to improve hearing outcomes for CI listeners.

## Bob Carlyon and John Deeks

### To What Extent are the Limits on CI Users’ Use of Purely Temporal Cues to Perceive the Pitch and Spatial Location of Sounds

#### (a) Due to a Fundamental Biological Limitation?

As noted in the Introduction, processing of fine timing cues by CI listeners is impaired even when the speech processor is bypassed and idealized stimuli are presented to a single electrode. The pitches of such stimuli rise with increases in pulse rate only up to some “upper limit,” which varies across listeners but is typically in the range 300–500 pps (e.g., Kong et al., 2009; Townshend et al., 1987; dashed line with arrows in Figure 2a). Similar findings have been observed with other periodic electrical stimuli such as sinusoids and amplitude-modulated (AM) pulse trains (Kong et al., 2009; Shannon, 1983). This contrasts with the situation in NH where many researchers believe that phase-locking to pure tones contributes to pitch perception up to at least 1000 Hz, with some arguing that it does so up to about 8000 Hz (Verschooten et al., 2019; but see Oxenham et al., 2011). Even for pulse rates of about 100–150 pps, where CI listeners’ detection of rate changes is best, difference limens (DLs) are typically in the range 5%–20%, considerably larger than the DLs of less than 1% for 125 Hz pure tones that have been reported in NH (e.g., Moore, 1973; see summary in Figure 2). Processing of ITDs also deteriorates at high rates (Figure 3); here we focus on pitch perception and refer the reader to the sections of this article that focus on ITD limitations.

A useful source of information on the nature of the neural limitations on temporal pitch perception in CIs comes from experiments with NH listeners, using acoustic pulse trains that have been band-pass filtered so that their frequency spectra contain only the high-numbered harmonics of the pulse rate that are “unresolved” by the peripheral auditory system. Such stimuli share some significant features with electric pulse trains presented to a CI electrode, including stimulation of mid-to-basal regions of the cochlea and that changes in pulse rate do not produce detectable changes in the place of excitation. Experiments that manipulated the relative amplitude or timing of different pulses have reported very similar effects of these manipulations on pitch for acoustic (NH) and electric (CI) stimuli (Carlyon et al., 2002; van Wieringen et al., 2003). We therefore believe that filtered acoustic pulse trains presented to NH listeners provide a good model of “optimal” perception of electric pulse trains presented to a CI, in the absence of damage to the auditory system or of auditory deprivation.

As illustrated by the purple bar in Figure 2, rate DLs for low-rate (e.g., 100 pps) acoustic pulse trains that have been bandpass filtered into a high-frequency region are roughly similar (5%–10%) to that observed for electric pulse trains presented to the best-performing CI listeners (Carlyon & Deeks, 2002; Moore & Carlyon, 2005), showing that at these rates the limitations in temporal sensitivity are not specific to electrical stimulation. The comparison between electric and acoustic pulse trains for the upper limit of pitch is complicated by the fact that, for high pulse rates, acoustic pulse trains can contain harmonics that are resolved by the auditory system, leading to the presence of place-of-excitation cues. This complication can be partially overcome by summing the harmonics of pulse trains in so-called alternating (“ALT”) phase, which produces pulse rates equal to double the F0. Macherey and Carlyon (2014) required NH listeners to pitch-rank bandpass-filtered harmonic complexes of different F0 s and summed in either sine- or ALT phase. Pitch ranks for the ALT-phase stimulus increased up to an F0 of 315 Hz, at which point the pitch was equal to that of a sine-phase complex with F0 = 630 Hz. This shows that the highest pitch that can be produced by purely temporal cues in NH hearing is at least 630 Hz. At higher F0 s pitch ranks for the ALT-phase stimulus may have been affected by basilar-membrane filtering, and so the true limit may or may not be higher. The highest upper limit that is observed for the best-performing CI listeners, which is of course unaffected by basilar-membrane filtering, is about 800–900 pps (Kong & Carlyon, 2010). Note however that although pitch may increase with increases in rate up to 800–900 pps, this does not mean that CI listeners hear a pitch equal to 800–900 Hz. Rather, it is possible (and indeed likely) that over some range the slope of the function relating pitch to pulse rate decreases but is not zero.

We conclude that, at least at low rates, the limitations on the temporal processing of the best-performing CI listeners broadly resemble that observed in NH listeners presented with analogous stimuli. Hence, the limitations are not specific to electrical stimulation *per se*. However, performance can vary substantially between listeners and even between electrodes in the same listener, suggesting that there are additional factors that can degrade temporal processing, which we now consider with particular reference to the upper limit.

An important fact is that the upper limit differs between electrodes in the same CI (e.g., Cosentino et al., 2016), thereby demonstrating that poor performance cannot be attributed to a general problem with pitch perception. This conclusion is bolstered by the findings of Ihlefeld et al. (2015), who measured ITD discrimination as a function of pulse rate for three place-pitch-matched pairs of electrodes in each of seven bilaterally implanted listeners. They also measured rate discrimination for each of the six electrodes. Performance on both tasks deteriorated with increasing rate, as expected. Importantly, performance (d’) on the ITD task could to some extent be predicted by the lower of the d’ scores for monaural rate discrimination in the corresponding electrodes in each ear. They concluded that the variation in upper limit of pitch across electrodes at least partially shares a basis with that for a non-pitch task, namely lateralization. An obvious locus for this variation could lie in between-electrode differences in the temporal fidelity of the AN response. However, there is also evidence that the upper limit can be limited by processing central to the AN. Carlyon and Deeks (2015) measured electrically evoked compound action potentials (ECAPs) and rate discrimination with the same stimuli and same group of CI participants, and presented a preliminary analysis showing that the timing of the ECAPs was sufficiently accurate to support rate discrimination even for rates above the upper limit and for which discrimination was at chance. This finding, obtained with human CI listeners who had been deaf for many years, is broadly consistent with the excellent AN phase-locking to electrical stimulation in recently deafened animals, as discussed in the sections by Volmer and Ohl, Delgutte and Chung, and Kral and Tillein.

#### (b) Modified by the Presence and Type of Electrical Stimulation That They Have Experienced?

Physiological data from animals, reviewed elsewhere in this article, suggests that the neural coding of pulse rate and of ITD can be significantly affected by auditory deprivation and by the presence and type of chronic electrical stimulation that has been provided, particularly so early in life. However, data on the effects of early stimulation on monaural temporal coding on humans are quite sparse and have not been studied with enough participants for firm conclusions to be drawn. For example, Busby et al. (1993) tested four patients who had been deafened before the age of 3 and four adult-deafened patients on a rate-discrimination task, and found that three of the adult-deafened patients performed better than the early-deafened patients but that one did not.

There are more data available on the effects of deprivation and stimulation in adulthood. Cosentino et al. (2016) reported a correlation between duration of deafness and the upper limit of temporal pitch in nine CI participants. This value of *N* is modest and so we combined data from six studies from our laboratory (total *N* = 50), all of which used the optimally efficient MidPoint Comparison (MPC) ranking procedure to estimate the upper limit of pitch, and for which we had also recorded the duration of deafness for each participant (Table 1). We then performed a univariate analysis of covariance with upper limit as the dependent variable, study as a fixed factor, and duration of deafness as co-variate. This allowed us to estimate the correlation between upper limit and duration of deafness whilst removing the across-study differences that may have arisen from variations in the stimuli, methods, and participants. The effect of study was significant, likely reflecting differences in the maximum pulse rate presented in the different studies (*F*[6,42] = 3.73, *p* = .005), but the effect of duration of deafness was not significant (*F*[1, 42] = 0.21, *p* = .65, *r* = −.07)). A similar analysis using age at testing as co-variate also failed to reveal an effect (*F*[1, 42] = 0.214, *p* = .65, *r* = −.07). Finally, we analyzed data on rate-discrimination thresholds and for a pulse rate close to 100 pps in five studies from different laboratories (total *N* = 45). Again there was a significant effect of study (*F*[5, 38] = 2.58; *p* = .042) but not of duration of deafness (*F*[1, 38] = 0.82, *p* = .37). When we analyzed the four studies where participant age was reported, the effect of age was once more not significant (*F*[1, 34] = 0.00, *p* = .98). Hence, we do not have any evidence that either the upper limit of temporal pitch or rate discrimination at low rates in adult CI listeners varies reliably as a function of the duration of deafness. It remains possible however that such an effect would have been observed in experiments designed to examine these factors and that therefore included a wider range of deafness durations and ages.

**Table 1.** Summary of Studies Included in the Meta-Analyses Described in the Section by Carlyon & Deeks.

		Correlation with upper limit	
Study	*N*	Deafness duration	Age at testing
Carlyon et al. (2010)	5	−0.83	−0.28
Carlyon et al. (2019)	9	0.21	−0.51
Carlyon et al. (2018)-Cochlear	7	0.45	0.61
Carlyon et al. (2018)-MedEl	5	0.47	−0.49
Cosentino et al. (2016)	9	−0.37	**−0**.**69**
de Groote et al. (2024)	8	0.36	−0.22
Lamping et al. (2020)	7	0.24	−0.18

(a) Correlation between the upper limit of pitch and both duration of deafness and age for each study. The sole correlation that was significant at the *p* < 0.05 level is shown in bold and did not survive correction for multiple comparisons. The study by Carlyon et al. (2018) was a longitudinal study of a pharmaceutical intervention; data from sessions 1 and 3 (baseline and post wash-out) were averaged and analyzed separately for the Cochlear and MedEl participants. Data from the study by de Groote et al. (2024) measured the upper limit for pulse trains applied to the most-apical electrode of the MedEl device. Data for the study by Lamping et al. (2020) were taken from the condition with monopolar stimuli presented to the most-apical electrode of the Advanced Bionics implant.

**Table 1.** continued

		Correlation with log (discrimination ratio)	
Study	*N*	Deafness duration	Age at testing
Stahl et al. (2016)	6	0.68	−0.38
Carlyon et al. (2018)	9	−0.27	0.62
Carlyon et al. (2018)-Cochlear	7	0.10	0.24
Carlyon et al. (2018)-MedEl	5	0.07	0.16
Lamping et al. (2020)	7	−0.32	−0.44
Goldsworthy et al. (2022)	12	**−0**.**55**	0.05

(b) Correlation between the logarithm of the rate discrimination ratio, for baseline rates between 80 and 120 pps, with duration of deafness and age. The sole correlation that was significant at the *p* < 0.05 level is shown in bold and did not survive correction for multiple comparisons. The study by Goldsworthy et al. (2022) included data for both ears of four listeners; the corresponding rate discrimination ratios were treated separately in the correlation with duration of deafness and averaged for the correlation with age. Data from Stahl et al. were averaged across the two electrodes studied and for a baseline rate of 104 pps. The study by Carlyon, Deeks et al. (2018) was analyzed as in part (a). Data from Lamping et al. (2020) were taken from Figure 8 of that paper for monopolar stimulation and an 80 pps baseline rate.

Correlational analyses are limited in that they cannot demonstrate causality. A longitudinal study by Carlyon et al. (2019) reported an increase in the upper limit between the day a participant's CI was first switched on compared to 2 months later. However, they noted that the stimulus level, equal to the most comfortable level at each session, had also increased, and so could not rule out the possibility that the improvement was due either to the increase in level or to practice. This latter issue is pertinent to another approach, adopted by Goldsworthy and colleagues (Bissmeyer et al., 2020; Goldsworthy & Shannon, 2014), who showed that rate discrimination by experienced CI listeners could be significantly improved by extensive training (Figure 2). Our view is that improvement with training has been reported for almost all tasks, can occur for many reasons (Ortiz & Wright, 2010), and does not necessarily reflect sensory plasticity. For example, participants may become more familiar with the experimental procedure, learn to use cues such as loudness or timbre that may co-vary with the temporal features of the stimulus, or become adept at “perceptual strategies” such as off-frequency listening in the measurement of psychophysical tuning curves observed in NH studies (Moore et al., 1984). Furthermore, if the especially poor rate discrimination at high rates (compared to lower rates) was due to speech processors failing to provide fast temporal fluctuations, then we might expect training effects to be larger at high than at low rates. This is because although CI speech processors do not preserve TFS, they do present pulse trains that are amplitude modulated at rates up to (but not beyond) a few hundred Hz (Figure 1b), and because listeners are sensitive to differences in those AM rates (e.g., Chatterjee & Oberzut, 2011). However, improvement on the rate discrimination task was either similar at all rates (Goldsworthy & Shannon, 2014; Figure 2a) or only significant at low pulse rates (Bissmeyer et al., 2020).

Finally, it is worth noting that variations in the upper limit and in ITD coding between electrodes, described above, reflect significant limitations on temporal processing that are unlikely to be driven by differences in exposure to CI processing strategies or to the duration of deafness, and that will likely limit the effectiveness of new attempts to improve sensitivity to these cues.

### What Would Change My Mind?

We are not convinced that there is evidence that training or long-term exposure to TFS cues in adulthood can improve temporal pitch processing. An important consideration when evaluating the effects of any manipulation, including training, on pitch perception is the nature of the psychophysical task. This is especially the case when, as with temporal pitch at high rates in CI listeners, the pitch percept is weak, and the participant may learn to perform the task using other cues. Procedures involving forced-choice tasks are likely to be susceptible to the use of extraneous cues when the same or similar standard stimulus is presented on every trial, when correct-answer feedback is provided, and/or an odd-one-out trial structure is employed. Procedures such as the MPC that do not provide feedback and where different pairs of stimuli are presented on each trial are less susceptible to these effects, and so improvements in the upper limit with training/exposure are less likely to be attributable to the use of extraneous cues. Even so, one cannot completely rule out practice effects and either of two other approaches would be needed to convince us that training or extended exposure in adulthood can genuinely improve temporal pitch processing. One would be a change in an objective measure of phase-locking, for example, the electrically evoked frequency following response (Gransier et al., 2024). The other would be a selective transfer of training—for example, showing that improvements in a rate discrimination task transferred more strongly to ITD discrimination than to monaural electrode discrimination in bilaterally implanted listeners.

### What If Anything Can Be Done to Improve the Temporal Processing of Pitch and Localization Cues by CI Listeners?

Although there are biological limits on temporal coding by CI listeners, it is also true that speech-processing strategies are far from optimized for pitch perception. Experimental approaches that enhance the modulations in each frequency channel and/or align the modulations across channels have produced modest but significant improvements in pitch perception with small groups of participants and may be worth more formal investigation in larger-scale trials (Francart et al., 2015; Lawrence, 1953; Milczynski et al., 2009; Vandali et al., 2019). Another approach, implemented commercially in some strategies, has been to present the TFS from the most-apical channels to the corresponding apical electrodes. This leads to different patterns of TFS being presented to each electrode, and so current spread could lead to apical neurons responding with a complex temporal pattern. We believe that a clearer pitch might emerge if the same pulse rate, perhaps derived from a real-time F0 tracker, was applied to these electrodes (de Groote et al., 2024).

Attempts to increase the upper limit and/or to improve pitch perception at low rates in CI listeners will depend on why performance is poor to begin with for these stimuli. One possible explanation arises from the observation that the traveling-wave delay for pure tones in NH is absent with electrical stimuli, and that the auditory system might “correct” for a delay that is not present with electrical stimulation. This would cause different parts of the excitation pattern to be processed with different delays, thereby blurring the temporal representation of each pulse (Šodan et al., 2024). If so, then stimulation methods that produce narrow excitation patterns might improve temporal coding. However, at least with existing technology, the maximum temporal processing expected from CI listeners is likely to be limited to that of NH listeners presented with analogous stimuli, which still falls short of that experienced by NH listeners in everyday situations. Finally, selective excitation of the apical AN might activate a pathway that is specialized for accurate temporal processing, as suggested by recordings from the cat inferior colliculus (IC; Middlebrooks & Snyder, 2010). Selective apical stimulation is unfortunately not available with existing CIs, because the only (MedEl) device that has an electrode array that reaches the apex only supports monopolar stimulation. Psychophysical studies that compared stimulation of the most-apical electrode of long MedEl arrays with more-basal stimulation reveal improved rate discrimination at low rates but no increase in the upper limit (de Groote et al., 2024; Stahl et al., 2016). A further limitation of traditional (intra-scalar) CIs in humans comes from the limited extent of Rosenthal's canal, meaning that the pattern of apical stimulation will significantly depend on the survival and trajectories of peripheral processes (Kalkman et al., 2014). However further investigations using electrodes that directly contact the AN in cats (Richardson et al., 2024) and humans (Adams & Lenarz, 2023) are currently in progress.

## Ray Goldsworthy

### To What Extent Are the Limits on CI Users’ Use of Purely Temporal Cues to Perceive the Pitch and Spatial Location of Sounds

#### (a) Due to a Fundamental Biological Limitation?

Pitch perception and sound localization vary widely across CI users. For example, people born deaf, who receive CIs after the age of three or 4 years, generally have just noticeable differences for pitch around 4 semitones (∼25%) (Zaltz et al., 2018). In contrast, many CI users with a history of normal hearing can discriminate pitches less than a semitone apart for a wide range of simple and complex sounds (Goldsworthy, 2015; Looi et al., 2012). This diversity in outcomes is consistent with animal studies of long- and short-term effects of deafness (Fallon et al., 2014a, 2014b). Specifically, severe abnormalities in the auditory pathways occur with early postnatal deprivation, whereas, these effects are reduced in mature animals with previous auditory experience (Hancock et al., 2010; Powell & Erulkar, 1962; Webster, 1983). Consequently, individual differences in hearing loss history affect the physiological limits of pitch and sound localization based purely on temporal cues.

The neural circuitry for temporal processing is exquisite. Golding and Oertel (2012) described how dendritic filtering of octopus cells of the cochlear nucleus (CN) compensates for traveling wave delays across AN fibers responding to broadband sounds. They also described how principal cells of the medial superior olive detect coincident activation of tuned neurons from the two ears through separate dendritic tufts. Pajevic et al. (2014) summarized how conduction velocity, mediated by myelin, provides an additional mechanism of activity-dependent nervous system plasticity. These mechanisms of temporal processing, dendritic filtering, and regulation of conduction time along axons are sensitive to auditory deprivation (Long et al., 2018). Thus, while temporal encoding of electrical stimulation is highly synchronized in the AN, it is likely that downstream processing is degraded by synaptic degeneration and myelin pathology. Critically, however, there is evidence that neuronal activity promotes oligodendrocyte progenitors, cell proliferation, and myelin formation along axons throughout the mammalian lifespan (Chapman & Hill, 2020; Sinclair et al., 2017; Williamson & Lyons, 2018). The extent to which stimulus-driven plasticity across the lifespan can overcome deficits caused by hearing loss and sensory deprivation is unknown.

#### (b) Modified by the Presence and Type of Electrical Stimulation That They Have Experienced?

There is evidence that the neural mechanisms that support temporal processing in the auditory system degenerate with sensory deprivation (Fallon et al., 2014b), but there is also evidence that experience-driven plasticity persists throughout the lifespan (Long et al., 2018; Seidl, 2014; Seidl et al., 2010; Sinclair et al., 2017). The extent to which electrical stimulation can modify the biological limits of pitch and localization based on temporal cues depends on the fidelity of the cues provided. Most CIs do not use stimulation timing to convey acoustic TFS (Goldsworthy, 2022; Goldsworthy & Bissmeyer, 2023; Svirsky, 2017). Consequently, there remains much uncertainty whether timing cues can be learned, or relearned, as a cue for pitch and localization.

##### My Estimate of Best Outcomes

A frequently discussed limit of timing cues for CIs is the upper limit of pitch based on stimulation rate. Many studies have reported that CI users weakly—or categorically cannot—hear pitch for pulse rates above 300 Hz (Carlyon et al., 2010; Shannon, 1983; Tong et al., 1982; Zeng, 2002). This upper limit strongly contrasts with the upper limit of usable TFS in normal hearing described by Verschooten et al. (2019). In that article, expert opinions of the upper limit of usable TFS in normal hearing ranged from 1500 Hz to 10 kHz. If any of those experts are correct, then an upper limit of 300 Hz for CI users is a considerable and unfortunate loss of information. I was a graduate student when I first learned that CI users could not hear pitch associated with pulse rates above 300 Hz. As a CI user, I immediately wanted to hear this for myself. After I received the training to perform such experiments, I started exploring my own limits of pitch based on pulse rate. When I first started, I could not discriminate between pulse rates above 300 Hz. I built a training procedure, described in Goldsworthy and Shannon (2014) to provide practice listening to pitch comparisons in a pulse-rate range centered on an individual's upper limit. That study found that CI users could improve their ability to rank pitch of pulse rates, and most of the participants attained just noticeable differences better than 3 semitones (∼20%) for pulse rates as high as 1760 Hz (A6 in the Western music tradition).

Because I am a CI user and a scientist leading studies of electrode psychophysics, I am in a unique position to describe the qualitative aspects of pulse-rate pitch. My implant, an N22 from Cochlear Corporation, has a technological upper limit around 3520 pps (A7). I routinely listen to pulse rates up to this technological limit when I am setting up new experiments. I can consistently discriminate pulse rates up to 3520 Hz with just noticeable differences of 2 semitones. I am often asked if the percept is pitch or some other percept. It is clearly pitch for pulse rates up to 440 Hz (A4), but the pitch salience diminishes between 440 and 880 Hz (A5), at which point the pitch is a weak, buzzy percept, but one that still allows distinction of pulse rates. Qualitatively, pulse rates above 880 Hz convey a sense of pitch height, but it is a weak percept. To make an estimate, the upper limit of temporal pitch in highly trained CI users is around 880 Hz with diminishing returns above that rate. Nevertheless, pulse rate provides a weak sense of pitch for rates as high as 3520 Hz (A7).

Perhaps more important than the upper limit is the corresponding lower limit of resolution. Studies have typically found that CI users can only discriminate rates that differ by 2 semitones or more (10%–20%) even for relatively low pulse rates between 100 and 300 Hz (Kong & Carlyon, 2010; Zeng, 2002). These lower limits of discrimination based on timing cues agree with observed limits in people with normal hearing listening to timing cues of varying temporal precision (Oxenham et al., 2004; Shackleton & Carlyon, 1994). Studies that use temporally less precise stimuli, such as AM sinusoids, typically find discrimination thresholds between two and three semitones (∼10%–20%), but those that use temporally precise acoustic pulse trains find thresholds of a half semitone (2%–3%) (Deeks et al., 2013; Kaernbach & Bering, 2001). Most studies of CI users find lower limits of resolution more like normal hearing for less precise timing cues (Kong & Carlyon, 2010; Zeng, 2002); however, there is evidence that CI users can take advantage of higher temporal precision provided by variable pulse rates compared to AM pulse trains (Baumann & Nobbe, 2006; Goldsworthy et al., 2021, 2022), and that discrimination for temporally precise stimuli improves to better than a semitone with training (Goldsworthy & Shannon, 2014). Given these considerations, I estimate that the lower limit of discrimination for CI users is about a half semitone (2%–3%) for pulse rates as high as 440 Hz.

Unlike pitch based on stimulation rate, I have no experience listening to interaural aural timing differences since I am unilaterally implanted with no residual hearing in my right ear, but the literature describes CI users as typically having just noticeable differences for interaural timing differences around 200 μs or worse for pulse trains presented to pitch-matched electrode pairs (Kan & Litovsky, 2015; Laback et al., 2015). This notably poor detection of interaural timing differences worsens with increasing pulse rate. There is an upper limit associated with increasing pulse rate and a lower limit of resolution within the usable range. The best outcomes reported in the literature indicate that CI users attain lower limits less than 100 μs for pulse rates as high as 1000 Hz (van Hoesel et al., 2009). This resolution observed in laboratory assessments of interaural timing sensitivity is remarkable given that clinical devices do not synchronize stimulation. Best outcomes for interaural timing discrimination might improve to better than 20 μs for pulse rates up to 1000 Hz once CI users are provided coordinated and synchronized bilateral stimulation.

### What Would Change My Mind?

To better characterize the limits of timing cues for pitch and sound localization, studies should provide CI users with timing cues in a clear and consistent manner while providing them extended exposure, familiarization, and training for these new cues. Deep longitudinal assessments, with participants followed over months and years, would characterize learning as participants approach their peak potential. This approach should incorporate engaging games to encourage attention and motivation for learning. A study that assesses learning of stimulation timing in a dozen participants, with timing cues precisely provided using psychophysical methods and synchronized hardware, with participants receiving hundreds of hours of familiarization and training, would demonstrate the extent that learning persists and thus could change my mind as to the limits of timing cues for pitch and localization.

Likewise, studies of new, fully implemented, stimulation strategies could also better characterize the limits of timing cues for pitch and localization. The problem with prior studies is that there is too much uncertainty as to how well new strategies encode timing cues. Future studies should provide better stimulation monitoring, for example, by recording stimulation patterns during everyday exposure. Similar tools already exist on clinical processors but with limited capacity. Future experiments could record daily stimulation as evidence that the new strategy does, in fact, encode timing cues with precision. A study that assesses pitch or localization with new stimulation strategies designed to encode acoustic TFS, while providing stronger evidence that the strategy effectively encodes timing cues, would demonstrate the limits of learning for these new cues, and thus could also change my mind.

### What if Anything Can Be Done to Improve the Temporal Processing of Pitch and Localization Cues by CI Listeners?

My central hypothesis is that CI users can learn to use timing cues for pitch and localization if these cues are provided in a clear and consistent manner. I believe that existing stimulation strategies for CIs do not provide these cues in a clear and consistent manner. Specifically, timing cues of nearby electrodes are smeared by current spread, thus degrading neural representation, which was also suggested by van Hoesel (2007). If current spread is a primary limitation for transmitting acoustic into neural representation of TFS, then there are both short- and long-term solutions. A short-term solution would be stimulation strategies modeled after peak-derived timing (PDT) or fine structure processing (FSP) but that provide relatively sparse spectral stimulation for electrode regions where temporal cues are most important (i.e., low frequencies). Unlike existing implementations of PDT and FSP, which attempt to convey TFS for all harmonics of a periodic sound, a spectrally sparse representation would provide a single place of stimulation across two or three electrodes with a covarying stimulation rate to represent fundamental frequency. There is evidence that CI users have better discrimination when stimulation place and timing cues covary than with either cue alone (Bissmeyer & Goldsworthy, 2022). Though there is evidence that this advantage may be a combination of independent cues rather than a dependent synergy (McKay et al., 2000), we note that for a dependent synergy to arise, the covaried place-rate stimulation would need to be consistently provided, thus affording the listener opportunity to learn (Keysers & Gazzola, 2014). The long-term solution may depend, ironically, on improving place precision of stimulation. The many efforts to improve place of stimulation using intraneural, magnetic, and optic stimulation, perhaps combined with neurotrophic support may lead to better specificity for place of excitation (Lee et al., 2022; Middlebrooks & Snyder, 2007; Moser & Dieter, 2020). In so doing, these solutions for providing better place of excitation might also provide independent neural channels for processing TFS.

The auditory system is justly celebrated for its remarkable tonotopic and temporal response properties. The importance of resolved harmonics for pitch and of low-frequency interaural timing differences for localization is clearly established. Many people might first think of place-of-excitation cues when considering resolved harmonics, but resolved harmonics also provide separate neural processing channels for TFS. Existing electrode arrays, combined with current spread in the cochlea, do not provide resolved place-of-excitation cues for densely spaced harmonics; consequently, they also do not provide separate processing channels for TFS for all harmonics of a complex sound. Recognizing this, I believe that stimulation strategies that provide focused delivery of the fundamental frequency of a complex sound into a clear and consistent combined place-rate stimulation cue have the best potential for improving both pitch and localization for CI users.

## Ruth Litovsky

### To What Extent are the Limits on CI Users’ Use of Purely Temporal Cues to Perceive the Pitch and Spatial Location of Sounds

#### (a) Due to a Fundamental Biological Limitation?

Individuals with normal hearing (NH) are known to utilize binaural cues to determine the location of a sound source in the horizontal plane and to distinguish target speech from background noise (Litovsky et al., 2021). These cues consist of ITDs and interaural level differences (ILDs). NH listeners typically have excellent sensitivity to both ITDs and ILDs, but they rely more heavily on ITDs at low frequencies to localize broadband sound sources (Blauert, 1996; Macpherson & Middlebrooks, 2002). Early studies in bilaterally implanted patients demonstrated that, while two CIs result in better spatial hearing abilities than unilateral CIs, performance seen in bilateral CI users is worse than performance of NH listeners. Various factors have been considered to contribute to this gap in performance, with temporal coding being one of the most significant culprits. Research findings discussed below suggest that temporal coding is impacted both by today's clinical CI speech processors, which are not designed to preserve finely controlled low-frequency ITDs, and by alterations to the auditory system due to deprivation during periods of deafness.

In bilateral CI users, clinical speech processors pose several notable limitations. Each CI processor is fitted independently to each ear, without any obligatory coordination or synchronization of inputs to the two ears. The term “synchronization” used here denotes the timing of sampling by the analog-to-digital converter and the timing of electrically pulsed stimulation delivered to specific electrodes in the right and left ears. A related issue is the actual limited encoding of binaural cues. First, if the processors are not simultaneously activated, a constant offset can occur between the two processors, ranging from −550 to +550 μs for stimulation rate of 900 pps as illustrated in Figure 1d and demonstrated by van Hoesel et al. (2002). Second, jittered timing between the processors in the two ears could occur due to the two processors having independent timing clocks that may drift over time. Third, some CI speech processing strategies (e.g., ACE) rely on “peak-picking” in which acoustic inputs are used to determine which set of channels are activated at each moment in time, and thus likely to have differences in channels at the two ears which minimize binaural cues (Kan et al., 2018). Even if these issues did not present limitations, signal processing strategies used in today's CI processors are inherently problematic. In general, TFS of the acoustic input is replaced with fixed-rate stimulation that is typically around 1000 pps—a rate that is too high for CI users to extract usable low-frequency ITDs (Laback et al., 2015; van Hoesel et al., 2009). The limitations serve as an important lens through which we can view the impact of experience with temporally coded inputs, as discussed further below.

Using research processors that bypass the clinical processors, researchers can electrically stimulate selected pairs of electrodes in the right and left ears. The unique nature of such studies is that electrode pairs are deliberately coordinated with precisely controlled timing to the two ears. Studies to date show enormous variability in sensitivity to ITDs across groups of bilateral CI users (Kan et al., 2013; Laback et al., 2015; Litovsky et al., 2012; Thakkar et al., 2020). This variability has been shown for various stimuli, with much of the data focusing on low stimulation rate of 100 pps, which is known to produce best performance, that is, lowest ITD discrimination thresholds. At 100 pps, the range of ITD thresholds found in adult bilateral CI listeners extends from a few tens of μs (within normal limits) to over 1000 μs (Cleary et al., 2022; Thakkar et al., 2020). The poor sensitivity of many CI listeners, even when presented with optimized stimuli, whereby the limitations imposed by the speech-processing strategy are bypassed, reflects a basic inability of the auditory system to process interaural timing cues. In the following section, I will argue that this reflects a basic biological limitation that arises from a combination of deprivation of binaural cues and exposure to suboptimal processing strategies.

#### (b) Modified by the Presence and Type of Electrical Stimulation They Have Experienced

A number of studies have shown that the across-listener variability can be attributed to age- and experience-related factors. The age at which onset of deafness occurs is an especially important factor; individuals whose auditory system has received normal acoustic input during development are more likely to retain sensitivity to ITDs than individuals who were deprived of acoustic hearing early in life. Litovsky et al. (2010) identified age at onset of deafness as a potential factor to consider, in a relatively small *N* size of patients. Thakkar et al. (2020) then measured sensitivity to ITDs and ILDs in the largest cohort known to date, 46 adult bilateral CI users who varied as to whether they had onset of deafness pre- or post-language acquisition. They found that binaural sensitivity was best in individuals who experienced shorter duration of bilateral hearing impairment, who had greater duration of experience with CIs, and who were younger at the time of testing. However, it is important to note that very few of these listeners show ITD sensitivity within the range of that observed in NH listeners. This is not surprising, given that in their daily lives bilateral CI users do not receive ITD cues with fidelity through their clinical processors. The impact of years of deprivation is also a likely factor. Notably, sensitivity to binaural cues is not affected uniformly—while ITD sensitivity in adults is clearly impacted and difficult to restore to CI users, sensitivity to ILDs might be less impacted (Litovsky et al., 2010; Thakkar et al., 2020). A deeper understanding of the extent to which ILD processing is impacted is needed, as some evidence suggests that even ILD processing is not on par with that of NH listeners, including both adults (Litovsky et al., 2010; Thakkar et al., 2020) and children (Easwar et al., 2017; Ehlers et al., 2017; Salloum et al., 2010).

Studies in children who are bilaterally implanted show that neural circuitry involved in binaural processing can fail to develop properly, as indicated by asymmetry of brainstem function shown by differences between the right and left auditory pathways in brainstem response latencies (Steel et al., 2015). Downstream effects on cortical asymmetries have also been shown (Lee et al., 2020; Polonenko et al., 2017). It is also possible that binaural neural circuits develop in early life but deteriorate after onset of deafness (Kral, 2013; Polonenko et al., 2018). Studies in animals that are deafened either neonatally or during early development have defined early periods involved in the maturation of auditory circuitry and pathways at the level of cellular morphology, molecular and synaptic properties, and tuning to ITD. Binaural processing depends on very precise timing of neural responses (spikes) from the left and right AN, in order for brainstem mechanisms to code ITDs with fidelity. Additionally, inhibitory synapses onto neurons in the brainstem are refined in substantial ways through synaptic and structural alterations during auditory development; importantly, these refinements in NH animals depend on auditory input and experience and are at risk for deterioration due to deafening (Kapfer et al., 2002; Werthat et al., 2008). Studies conducted in animals who receive CIs also suggest that binaural processing is impacted by degraded balance of inhibitory and excitatory inputs, and is associated with poor tuning of neuronal ITD properties in the auditory brainstem and cortex in deafened, implanted animals (Chung et al., 2016; Hancock et al., 2013; Jakob et al., 2019; Tillien et al., 2010). To date, little is known about how such disruptions and alterations are controlled or prevented. It stands to reason that in humans who are deaf and deprived of access to acoustic hearing, the neural mechanisms involved in processing ITD cues may be at risk for permanent disruption with limited potential for restoring processing to NH levels of functioning. This problem is intricately related to the fact that, if children grow up with bilateral CIs that fail to deliver low-rate, synchronized, and well-preserved ITDs, their auditory system is likely to eventually lose the capacity to have sensitivity to ITDs restored with future generation of signal processing strategies that provide access ITDs. Furthermore, even in one ear alone, encoding of TFS in electrical stimulation by CI users is limited by stimulation rates that are higher than about 300 pps. There is an extensive literature (covered elsewhere in this paper) discussing the problems with sensitivity to temporal properties of electrical stimulation, which deteriorates at much lower rates than seen in NH listeners. Rate limitations were shown in monaural stimulation (e.g., Carlyon et al., 2008; Kong et al., 2009; Kong & Carlyon, 2010; McDermott & McKay, 1997; Shannon, 1983; Zeng, 2002) and in binaural stimulation (Carlyon et al., 2008; van Hoesel 2007; van Hoesel et al., 2009). Critically, there is evidence to suggest that monaural rate sensitivity and binaural sensitivity for ITDs may be limited by a shared mechanism (Ihlefeld et al., 2015). The extent to which the shared mechanism reflects information transmission, health of neural elements, or integrity of electrode–neuron interface remains to be determined.

### What Would Change My Mind?

Thus far, this section has focused on how limitations in today's CIs limit the ability of bilateral CI users to fully benefit from binaural hearing. For children, the greatest risk is disruption to the neural mechanisms involved in ITD processing and downstream effects on central processing of binaural information. My mind regarding this limitation would change if data suggested that infants and young children who are exposed to ITDs early in life do not achieve the same level of performance as peers with NH. That outcome would likely occur if electrical stimulation cannot achieve the same type of processing as acoustic stimulation and/or if the underlying neural infrastructure of deaf infants and children is differently wired and simply cannot decode and encode low-frequency ITDs in such a way that provides benefits observed in NH listeners. Such a finding would potentially place stronger pressures on advancement of genetic testing and biologically based treatment for deafness with approaches such as gene therapy and/or regeneration.

### What, If Anything, Can Be Done to Improve CI Performance for Pitch and Binaural Time Processing?

The clearest potential approach to modifying and improving temporal coding by experience in childhood is to ensure that infants who are deaf and are implanted with bilateral CI devices can receive binaural cues with fidelity. That will mean engineering bilaterally synchronized devices that operate successfully in everyday environments. The devices must be able to minimally (1) capture binaural cues at multiple frequency channels, (2) preserve low-frequency onset and ongoing ITDs with precision known to occur in normal acoustic hearing, (3) preserve speech envelope cues in at least some of the channels, and (4) process multi-source information and reverberation. One possibility is the CCi-MOBILE device, which is a portable research device compatible with Cochlear Ltd. (Ghosh et al., 2022; Hansen et al., 2019), with potential to be extended to other CI manufacturers. The CCi-MOBILE is bilaterally synchronized; thus, it operates using a single time clock to simultaneously extract information from two microphones and deliver coordinated stimulation to two CI processors. The CCi-MOBILE is the only portable research processor that can operate without being tethered to a computer, and that is capable of real-time processing, with the potential to account for the hardware limitations described above. The device has been recently implemented with the use of envelope ITDs (Dennison et al., 2023) and has the potential to be further developed for coding TFS and very small ITDs at low frequencies. If these devices are not available in clinical applications, perhaps an interim step can be taken to offer take-home devices that allow listening through computer interfaces to stimuli that are processed with binaurally preserved cues. That would minimally provide the developing auditory system with daily exposure to the information that is needed for optimal coding of temporal cues. Such an approach will need to be investigated in clinical trials, with outcome measures that focus not only on temporal coding and binaural sensitivity, but downstream effects on cognitive abilities, listening effort, and more generalized aspects of speech understanding and language development. If these “interim” interventions were available, they could be used to prepare the brain to take advantage of improved temporal coding and binaural processing when those processors become clinically implementable.

The ultimate solution is to promote reengineering of CIs such that signal processing encodes and transmits TFS cues with fidelity, while preserving speech envelope cues. The general idea is to convey ITDs in the timing of electrical pulses on some channels by firing them at low stimulation rates but preserving high rates at other electrodes. By sending low-rate stimulation to some electrodes, and high-rate stimulation to other electrodes, it may be possible to transmit ITDs at the low rates and to preserve speech envelope cues at electrodes receiving high rates. Over the years, approaches included the PDT strategy (van Hoesel, 2007), the FSP/FS4 strategy which is designed to slow down the repetition rate to follow the instantaneous TFS frequency by introducing a pulse at each positive-going zero crossing in the bandpass filter output of a channel (Hochmair et al., 2006; Zirn et al., 2016); these strategies were not yet been shown to benefit bilateral CI users. Litovsky et al. have been testing a mixed-rate strategy approach which deliberately sends low-rate stimulation to interaural pairs of electrodes that are pre-tested based on knowledge that they produce good ITD sensitivity, and high rates to other electrodes to preserve speech cues (Thakkar et al., 2018, 2023). This approach has shown promising results thus far for preserving ITD sensitivity, but its efficacy for preserving speech cues remains to be seen. Another approach, a temporal limits encoder strategy (Zhou et al., 2022) was suggested as a means of improving pitch discrimination and tone recognition in languages such as Mandarin (Zhou et al., 2022). By down-transposing mid-frequency channel information at restricted bands to lower frequencies, envelope modulations are slowed down, and when used bilaterally, this strategy has the potential to encode ITDs within the down-transposed envelope modulations (Kan & Meng, 2021). Again, studies to date have shown modest outcomes, in bilateral CI listeners. Importantly, while in acoustic hearing low-frequency ITDs are known to be processed in the apical region of the cochlea, when selecting electrodes for delivery of temporal information such as ITDs, stimulation need not be presented to apical electrodes; in fact, mid- or basal-stimulation can produce best ITD sensitivity in many listeners (Litovsky et al., 2010, 2012; Thakkar et al., 2020). This idea is critical in the future design of novel stimulation strategies, as it must consider the fact that neural health varies along the electrode arrays in each ear, and across individual CI users. The variation is a complex product of effects of auditory deprivation, trauma, and many factors that impact survival and function of the AN, as well as neural processes at the brainstem and beyond.

#### Could Performance in CI Theoretically Match That of NH Listeners One Day?

Theoretically, yes! The key factor is providing CI users with stimulation that mimics acoustic hearing as much as possible. If signal processing strategies can be designed as such, then infants and children who are congenitally deaf or children who would receive appropriate binaural and pitch cues from a young age have the potential to experience auditory development on par with that of NH listeners. Adults who experience deafness after they have already developed a normal auditory system through acoustic hearing would then benefit from the same improved engineering processes that are akin to inputs enjoyed by NH listeners.

## Bertrand Delgutte and Yoojin Chung

### To What Extent Are the Limits on CI Users’ Use of Purely Temporal Cues to Perceive the Pitch and Spatial Location of Sounds

#### (a) Due to a Fundamental Biological Limitation?

The deficits in the perception of temporal cues in users of CIs are due to fundamental neural limitations that are influenced by auditory experience, especially during development. These limitations are not primarily of peripheral origin, but rather result from a reduced ability of the central processor to make effective use of the temporal cues delivered in the ANs. Our opinion derives from comparing data on responses of auditory neurons to electric stimulation in animal models of CIs with perceptual results in human CI users. We primarily discuss responses to constant-amplitude, electric pulse trains, which are the simplest stimuli for understanding fundamental limitations on temporal processing.

##### Exaggerated Temporal Coding in the Auditory Nerve with Electric Stimulation

In NH animals, AN fibers phase lock to sinusoidal acoustic stimuli (pure tones) for frequencies up to 3–5 kHz (Johnson, 1980; Palmer & Russell, 1986), and this limit is probably no higher in humans (Verschooten et al., 2018). In contrast, AN fibers phase lock to sinusoidal electric stimuli up to at least 10 kHz, more than an octave higher than for acoustic stimuli (Dynes & Delgutte, 1992; Hartmann et al., 1984). For pulse-train stimuli like those used in most CI processors, synchronization to pulse rates as high as 5000 pps has been reported in cat AN fibers (Miller et al., 2008). Moreover, AN fibers can entrain (fire one synchronized spike per pulse) to electric pulse trains for rates up to 800 pps, much higher than for acoustic stimulation (Javel & Shepherd, 2000; Shepherd & Javel, 1997).

The very high limit of synchronization of AN firings to electric pulse trains observed in experimental animals also applies to human CI users. The ECAP of the human AN has been isolated in response to individual pulses in a pulse train for rates as high as 3500–4000 pps (Hughes et al., 2012; Tejani et al., 2017). The presence of such synchronized responses in the ECAP implies not only that a large number of AN fibers synchronize to the pulse train, consistent with the single-fiber recordings in animals, but also that the AN firings are synchronized to each other (“across-fiber synchrony”). The precise synchrony of AN fibers is observed not only for constant-amplitude pulse trains, but also to the modulation waveform of AM pulse trains, in both animals (Jeng et al., 2009) and human CI users (Tejani et al., 2017). The exaggerated synchrony observed in the AN with electric stimulation also holds for neurons in the anteroventral CN and the medial nucleus of the trapezoid body (MNTB), two auditory brainstem nuclei involved in binaural processing (Müller et al., 2023). These results suggest that the perceptual limits on rate pitch (Carlyon & Deeks, 2002; Kong et al., 2009) and binaural interactions (Laback et al., 2015) with CIs are not caused by a lack of precise temporal information in the peripheral inputs.

The massive across-fiber synchrony occurring with electric stimulation results from several factors, including: (1) the spatial patterns of AN excitation along the tonotopic axis of the cochlea are broader for electric stimulation than for pure tone stimuli; (2) the cochlear traveling wave disperses the latencies of AN fibers to broadband acoustic stimuli such as clicks, while there is no traveling wave with electric stimulation; (3) AN fibers fire to electric stimuli more deterministically (less stochastically) than they do for acoustic stimuli (Kiang & Moxon, 1972). The massive across-fiber synchronization in electric stimulation may impair the ability of central circuits for binaural (ITD) and pitch processing to make use of the available temporal information.

##### Central Limitations on ITD Sensitivity with CIs

The initial stages of ITD processing in the lateral and medial superior olives (LSO and MSO) in NH animals are relatively well understood (Grothe & Pecka, 2014; Joris & van der Heijden, 2019; Yin et al., 2019). Unfortunately, no study has yet recorded responses of MSO and LSO neurons to bilateral electric stimulation through CIs; the available data are mostly from the IC in the midbrain, to which both LSO and MSO project. MSO and LSO neurons transform interaural differences in the timing of their spike inputs into changes in firing rate via a process of coincidence detection (or anticoincidence for LSO). Following this transformation, ITDs are primarily represented by a rate code rather than a temporal code in the IC and beyond (Fitzpatrick et al., 1997, 2000, 2002).

For optimal stimulus conditions, many IC neurons in acutely deafened animals are sensitive to ITDs of bilateral electric pulse trains, in proportions comparable to those observed for broadband acoustic stimuli in NH animals, and the shapes of ITD tuning curves resemble those observed for acoustic stimulation (Chung et al., 2016; Smith & Delgutte, 2007; Sunwoo & Oh, 2022; Vollmer, 2018). However, good ITD sensitivity only occurs over a narrow range of pulse rates and stimulus levels. For most IC neurons in anesthetized preparations, ITD sensitivity is limited to the onset of electric pulse trains for pulse rates above 100 pps (Smith & Delgutte, 2007; Sunwoo & Oh, 2022). Sensitivity to ongoing ITDs at higher pulse rates is more common in the IC of unanesthetized animals (Chung et al., 2016) and in neurons that respond to apical stimulation of the cochlea (Sunwoo & Oh, 2022). The rate limitations observed in neural responses to electric stimulation are consistent with the low limit of perceptual ITD sensitivity in CI users (Laback et al., 2015) but contrast with the responses to isolated pairs of binaural pulses reported in the rat (Buck et al., 2021; see section by Schnupp & Rosskothen-Kuhl). These low limits contrast with the ∼2000 Hz limit of neural ITD sensitivity to the ongoing temporal time structure in NH animals (Devore & Delgutte, 2010; Joris, 2003; Yin & Kuwada, 1983) and the ∼1400 Hz limit of perceptual ITD sensitivity in human NH listeners (Brughera et al., 2013).

It has been suggested that the poor ITD sensitivity with CIs results from a switch from MSO dominance in NH to LSO dominance with CI because CI devices may not reach sufficiently far into the cochlear apex to stimulate the MSO neurons, which are tuned primarily to low frequencies (e.g., Dietz, 2016; Müller et al., 2023). A further argument for this view is that perceptual ITD sensitivity in bilateral CI users is more in line with the sensitivity to envelope ITDs thought to be created in the LSO than with the sensitivity to ITDs in the TFS created in the MSO. While this view has the appeal of simplicity, it fails to account for important observations. Many IC neurons in deaf animals show peak-type ITD tuning for electric stimulation similar to the tuning of MSO neurons in NH animals (Chung et al., 2016; Smith & Delgutte, 2007; Sunwoo & Oh, 2022), and many neurons sensitive to ITD with electric stimulation in a preparation with preserved hearing are tuned to low acoustic frequencies in the range of MSO neurons (Vollmer, 2018). Whole-cell recordings in NH animals show that, contrary to the view that LSO neurons are sluggish compared to MSO neurons (Remme et al., 2014), LSO principal cells in fact display better ITD sensitivity than MSO neurons for click stimuli resembling the pulses used for electric stimulation (Franken et al., 2021). Thus, the low rate limit of ITD sensitivity with electric stimulation may not be caused by a failure to effectively stimulate the MSO circuit with CIs, but rather by a degraded sensitivity in both LSO and MSO.

We suggest that the abnormally broad spatial patterns of excitation and the excessive across-fiber synchrony produced in the AN by electric stimulation may engage inhibitory and other suppressive mechanisms in the brainstem more effectively than acoustic stimulation, thereby blocking excitatory responses at high pulse rates. Excessive synchrony in the inputs to MSO may also increase monaural coincidences, leading to degraded ITD sensitivity (Chung et al., 2015).

##### Central Limitations on the Coding of Temporal Pitch with CIs

In NH animals, the ability of central auditory neurons to phase lock to either the TFS or the temporal envelope of acoustic stimuli tends to degrade as one ascends the auditory pathway (Joris et al., 2004; Liu et al., 2006). As the temporal code degrades in the IC and beyond, the repetition rate of acoustic stimuli is increasingly represented by a rate code, whereby the firing rates are tuned to the envelope repetition rate of AM tones and harmonic complex tones (Joris et al., 2004; Lu et al., 2001; Nelson & Carney, 2007; Su & Delgutte, 2019). In principle, the stimulus repetition rate can be decoded from the across-neuron pattern of firing rates in a population of neurons whose firing rates are tuned to different repetition rates.

Both the temporal code (Chung et al., 2014; Hancock et al., 2013; Middlebrooks & Snyder, 2010; Snyder et al., 1995, 2000; Vollmer et al., 1999, 2005) and the rate code (Chung et al., 2014; Hancock et al., 2012; Snyder et al., 1995) are also present for electric pulse trains in the IC of implanted animals, but the two codes are subject to different limitations.

Su et al. (2021) directly compared the limits of synchronization to electric pulse trains in the IC of unanesthetized rabbits with the limits for the most comparable acoustic stimulus, a click train. The synchronization limits were higher for electric stimuli (median 206 pps) than for acoustic stimuli (112 pps). Thus, in the IC like in the AN, performance with CIs is not limited by a reduced availability of temporal information in the neural firing patterns. Importantly, in contrast to the temporal code, a lower range of pulse rates was represented by the rate code in the IC of CI animals compared to NH animals.

In most IC neurons, the limit of synchronization to electric pulse trains is lower than the ∼300 pps limit of temporal pitch perception in most human CI users (Carlyon & Deeks, 2002; Kong et al., 2009), suggesting that pitch perception at higher pulse rates is likely to rely on the rate code. The degradation in rate coding observed in the IC with electric stimulation is consistent with the lower limit of rate pitch perception for CI users compared to NH listeners. Still, the distribution of synchronization limits across the neuronal IC population is quite broad, so that some neurons still synchronize to pulse trains at 300 pps, and these synchronized neurons are particularly common in the IC region responsive to stimulation of the cochlear apex (Middlebrooks & Snyder, 2010).

The rate code to repetition rate is even more important in the ACx, where the limits of neural synchronization to electric pulse trains (Beitel et al., 2011; Fallon et al., 2014b; Johnson et al., 2017; Vollmer & Beitel, 2011), are much lower than in the IC, and below the range over which periodic pulse trains evoke pitch percepts.

The mechanisms for the transformation from a temporal code in the auditory periphery to rate codes in the IC and above are not fully understood. In one model, bandpass rate tuning is created via the interaction of fast excitation and slower, delayed inhibition (Hancock et al., 2017; Nelson & Carney, 2004; Smith & Delgutte, 2008). If so, the degradation in rate coding observed in the IC of deaf animals with CIs is consistent with the disrupted inhibition associated with hearing loss (Takesian et al., 2009).

Unlike ITD sensitivity, which is ultimately limited by the temporal windows of coincidence detection in LSO and MSO, the limitations on the coding of rate pitch are likely to arise in the IC and beyond where the transformations from a temporal code to a rate code take place. Because some of the inputs to the IC bypass MSO and LSO, the limitations on rate pitch may differ from those on ITD sensitivity. Consistent with this hypothesis, a new analysis of the data from Sunwoo et al. (2021) reveals no across-neurons correlation between the upper frequency limits of ITD sensitivity and synchronization to pulse trains in the IC of deaf rabbits. This result contrasts with the finding of a correlation between performance in monaural rate discrimination and performance in ITD discrimination in a modest number of CI subjects (Ihlefeld et al., 2015).

#### (b) Modified by the Presence and Type of Electrical Stimulation That They Have Experienced?

##### ITD Sensitivity

Deafness history and auditory experience with CIs can influence ITD sensitivity with bilateral electrical stimulation. A decreased incidence of ITD-sensitive units and poorer ITD sensitivity was observed in the IC of congenitally deaf cats (Hancock et al., 2010), and in neonatally deafened (ND) rabbits (Chung et al., 2019), cats (Thompson et al., 2021), and rats (Sunwoo, 2023) compared to animals with normal hearing during development. Similar trends have been observed in the auditory cortex (ACx) of congenitally deaf cats (Tillein et al., 2010). Modest improvements in ITD sensitivity have been reported in neonatally deafened animals that were provided with ITD cues through bilateral CIs during development (Sunwoo et al., 2021; Thompson et al., 2021).

Contrary to these trends, Rosskothen-Kuhl et al. (2021) reported behavioral ITD sensitivity comparable to normal in neonatally deafened rats that were implanted in adult age. (The neurophysiological results also presented in this paper cannot be meaningfully compared with those from other studies because they used single electric pulses rather than pulse trains.) This result contrasts with the poor perceptual ITD sensitivity in prelingually deaf human bilateral CI listeners (Ehlers et al., 2017; Laback et al., 2015). The authors suggest that maladaptive plasticity to conventional continuous interleaved sampling (CIS) processing might cause the poor perceptual ITD sensitivity in CI listeners. While this view may be plausible for prelingually deaf CI users, it cannot explain the poor ITD sensitivity in subjects with normal auditory development who became deaf in adulthood. To directly test this hypothesis, ITD sensitivity should be compared between unstimulated adult-deafened animals and animals given stimulation lacking ITD cues.

##### Pitch Processing

The limits of neural synchronization to electric pulse trains in the IC and ACx of CI animals are also influenced by deafness history (Hancock et al., 2013; Vollmer et al., 2005) and auditory experience with the CI (Snyder et al., 1995; Vollmer et al., 1999, 2005, 2017a), but the effects in the IC are modest. These studies were focused on temporal coding and did not analyze effects on the rate code which is likely to be important at higher pulse rates.

### What Would Change Your Mind?

Since we poorly understand how the abnormal spatiotemporal patterns of peripheral activity with CI may result in poor temporal and ITD coding by central neurons, new experiments are needed to unravel these mechanisms. Direct recordings from the primary sites of binaural interaction in LSO and MSO are needed to test the hypothesis that the deficits result from ineffective stimulation of the MSO. Alternatively, recordings from the IC and ACx, combined with selective manipulation of neural activity in LSO and MSO by optogenetic or pharmacological techniques, would be valuable. More studies are also needed to compare temporal coding and ITD sensitivity between CI and NH in the same species and using the same methods and comparable stimuli. These include studies using electric stimulation in animals with preserved hearing, so that the same neurons can be studied with both forms of stimulation.

Regarding the effects of deafness history and auditory experience with CIs on temporal coding, a major problem is that we don’t understand how the observed changes in neural activity impact behavioral performance. We need experiments combining neural recordings and measurements of behavioral performance in temporal tasks in the same animals, preferably performed simultaneously. Some of these experiments should use techniques such as two photon imaging and large-scale recording electrode arrays, to display the activity of large neuronal populations on which behavior is presumably based. In studies of the effect of experience with CIs on neural activity, chronic recording techniques allowing longitudinal experiments on the same set of neurons are needed to overcome the problem of large interneuron variability in studies that compared data from different groups of animals.

### What Can be Done to Improve CI Performance for Pitch and Binaural Time Processing?

If the deficits in temporal coding and ITD sensitivity primarily result from the inability of central circuits to efficiently process the abnormal spatio-temporal patterns of peripheral activity produced by electric stimulation, then technologies that achieve more-selective and less-synchronized patterns of stimulation should improve temporal pitch perception and ITD sensitivity in CI users. These technologies may include intraneural stimulation (Middlebrooks & Snyder, 2007), optical (Dieter et al., 2020) and magnetic (Lee et al., 2022) stimulation of the AN, and regenerative technologies to regrow AN fibers into the cochlea (Pinyon et al., 2019). We are less optimistic about further improvements from new processing strategies, although more work on TFS strategies for binaural hearing is needed. Finally, if the plasticity effects observed in neural recordings prove to have behavioral consequences, then more immersive training protocols in temporal tasks may prove effective in improving performance.

## Maike Vollmer and Frank W. Ohl

### To What Extent are the Limits on CI Users’ Use of Purely Temporal Cues to Perceive the Pitch and Spatial Location of Sounds Due to

#### (a) A Fundamental Biological Limitation?

Designing experiments allowing inference on neuronal processing of “purely temporal” cues poses challenges. Changes in the temporal properties of a stimulus affect both its temporal and spectral characteristics, and any process and mechanism characterized with reference to its spectral properties will be affected accordingly. For instance, increasing the rate of electric pulse trains in CI stimulation may not only reduce a neuron's threshold but also broaden the spatial pattern of activation of AN fibers along the tonotopic axis of the cochlea. This broadening cannot be compensated for by level adjustments based on psychophysical or electrophysiological measures without impacting temporal properties. These covarying aspects can fundamentally alter the encoding and perception of both pitch and spatial location.

Another challenge arises from the diversity of research strategies employed in investigating the deaf auditory system. Various factors, including species differences, deafening procedures, onset and duration of deafness, electrode location, stimulus properties, psychoacoustic task specifics, and data analyses, potentially influence quantitative and qualitative study results and hinder their comparisons and interpretation.

In the following, we will focus on limitations in the temporal precision of neuronal responses in the context of both monaural and binaural rate discrimination and ITD coding in response to electric stimulation. Specifically, we will concentrate on the encoding of TFS of electric stimulation using constant-amplitude periodic pulse trains of varying rates. It is noteworthy that although ITD sensitivity can be assessed with different types of stimuli (e.g., single pulses, periodic pulse trains, and pulse trains with irregular interpulse intervals), it is largely unclear how factors determining jitter in pulse-evoked timing of a neural response to an isolated pulse are modified in the context of additional pulses, their rate, and regularity. There is a general lack of studies addressing the occurrence and role of response jitter in these scenarios, not only concerning electric stimulation, but also with respect to acoustic stimulation in the healthy system.

Understanding the response properties of neurons at the primary sites of binaural interaction in auditory brainstem, namely the MSO and the LSO, as well as in the auditory midbrain (IC), requires characterization of the temporal firing properties in response to monaural and binaural inputs. In many mammalian species, AN fibers exhibit phase-locking to the TFS of a sinusoidal acoustic signal (pure tone) up to 5 kHz, although the degree of which is already deteriorating at ∼1 kHz (Johnson, 1980; Palmer and Russell, 1986). Notably, at the AN level, the upper rate-limits of phase-locking to sinusoidal electric (at least 10 kHz) and pulsatile electric stimulation (up to 5000 pps) can surpass that to acoustic stimulation (e.g., Dynes and Delgutte, 1992; Hartmann et al., 1984; Miller et al., 2008; Shepherd and Javel, 1997). At subsequent processing stages, namely the CN and the MNTB, the precision of temporal phase-locking to tones increases relative to that of ANs, at least for acoustic frequencies <1 kHz (e.g., Joris et al., 1994; Wei et al., 2023). At the MSO, where excitatory and inhibitory inputs from both ears converge, precise synchronization to the monaural inputs from both sides is crucial for the computation of ITDs and exists for acoustic (and presumably also for electric) stimulation. However, the upper limits of stimulation rates to which phase-locking occurs gradually decrease along the auditory pathway (e.g., CN: Blackburn and Sachs, 1989; Frisina et al., 1990; Rhode and Greenberg, 1994; MNTB: e.g., Bartlett and Wang, 2007; de Ribaupierre et al., 1980; Preuss and Müller-Preuss, 1990; Rouiller et al., 1981; IC: Batra et al., 1989; Krishna and Semple, 2000; Langner and Schreiner, 1988; Liu et al., 2006; Müller-Preuss et al., 1994; ACx: e.g., de Ribaupierre et al., 1972; Eggermont, 1991; Lu and Wang, 2000; Wallace et al., 2002). At the level of the IC, neural phase-locking to monaural electric pulse trains shows median upper limits of ∼100 pps in anesthetized preparations (e.g., cat: Vollmer et al., 1999) and ∼200 pps in awake preparations (e.g., rabbit: Su et al., 2021). However, the maximum upper limits of neural phase-locking to electric pulses in the IC extend to ∼300 pps or even higher (Hancock et al., 2013; Middlebrooks and Snyder, 2010; Su et al., 2021; Sunwoo et al., 2021; Vollmer et al., 1999, 2005), roughly corresponding to the upper limits of neural ITD sensitivity to electric pulses (e.g., Chung et al., 2016; Hancock et al., 2013). Moreover, these limitations in neural phase-locking to electric pulses are similar to the perceptual limits of rate discrimination in response to monaural periodic pulse trains in most CI subjects (∼300 pps, extending up to 900 pps in star subjects; e.g., Kong et al., 2009; Moore and Carlyon, 2005; Townshend et al., 1987; Zeng, 2002) and to their perceptual limits of ITD sensitivity (∼300 pps) (Ihlefeld et al., 2015; Kan and Litovsky, 2015; Laback et al., 2007, 2015; van Hoesel, 2007). Collectively, these results from animal and human studies suggest similar upper-rate limits for neural phase-locking, psychophysical rate discrimination, and both neural and psychophysical ITD sensitivities to electric pulse trains. However, across-neuron comparisons in rabbit IC showed no correlation between the upper rate limits of neural phase-locking and ITD sensitivity (see Sunwoo et al., 2021, and section by Delgutte & Chung).

Comparisons of limitations in rate discrimination and ITD sensitivity between CI subjects and NH listeners are hampered by differences in the temporal and spectral properties of the electric and acoustic signals. For example, the center frequency, bandwidth, rate, and sharpness of envelope fluctuations affect acoustic ITD sensitivity. Therefore, the finding that the upper limits of neural ITD sensitivity and perceptual ITD sensitivity to pure tones in NH animals and human listeners extends to ∼1400 Hz acoustic sinusoids (Brughera et al., 2013; Vollmer, 2018) does not automatically apply to other types of stimuli. To identify intrinsic differences in temporal coding between electric and acoustic stimulation, human studies have used trains of brief, bandlimited acoustic clicks (e.g., Carlyon et al., 2002; Kan et al., 2013; Majdak and Laback, 2009; McKay and Carlyon, 1999) to more closely resemble electric pulsatile stimulation in CIs. In CI listeners, the rate limitation for ITD discrimination varied between 100 and 800 pps. Generally, the average rate limits (∼300 pps) were similar for both human NH and CI listeners, but data from CI listeners showed a larger variability (e.g., Majdak and Laback, 2009; van Hoesel, 2007). Studies in NH and CI animals also compared ITD coding in response to electric pulses and transient acoustic stimuli (clicks, chirps; Su et al., 2021; Vollmer, 2018). At least for low stimulation rates, ITD discrimination thresholds and ITD tuning properties measured in the same neurons of CI animals with preserved hearing did not significantly differ between electric and acoustic stimulation (Vollmer, 2018). Another study comparing NH and deafened CI animals reported that the median and upper-limit stimulus rates that elicited maximum firing (“rate coding”) of IC neurons to electric pulses were lower than those to acoustic clicks (e.g., Su et al., 2021). However, due to the broad distributions of neural pulse-locking limits (“temporal coding”), a minority of neurons in both NH and CI animals synchronized to click and pulse rates, respectively, of 300 pps or above. At this point, it is not known whether median upper limits or maximum upper limits of rate coding or pulse-locking are more relevant for determining the upper rate limit of ITD coding.

Overall, results from both human and animal studies suggest that the (low) upper limits in rate discrimination and ITD sensitivity are not generally attributable to the electric nature of the stimulation. Rather, we argue that electric and acoustic stimuli with comparable temporal and spectral properties exhibit similar temporal limitations in rate following and ITD processing. The detailed mechanisms underlying the upper limits for rate discrimination and ITD sensitivity in response to electric pulse and acoustic click trains are not completely understood. It is possible that the spatially broad and highly synchronized response patterns to transient electric and acoustic stimuli are more effective in engaging inhibitory connections in the auditory brainstem than responses to spatially more-restricted and temporally more-dispersed acoustic stimuli and, thus, more strongly counteract excitatory responses at high rates of stimulation. When compared to clicks, we speculate that acoustic stimuli that even closer approximate the spatiotemporal response profile in AN fibers evoked by electric pulses—such as chirps with increasing instantaneous frequency (up-chirps), designed to compensate for spatial dispersion along the cochlea (e.g., Adel et al., 2021; Dau et al., 2000)—could result in more synchronized responses across the tonotopic axis and may further diminish the differences observed in temporal processing between electric and acoustic stimulation at higher rates.

In addition to electric stimulus properties, deafness-induced degradations can affect rate discrimination and ITD sensitivity in CI subjects. Performing a meta-analysis, Carlyon & Deeks (this article) found no significant correlation between deafness duration or age at deafness onset and the upper limit of rate discrimination in human CI users with adult-onset deafness. However, studies in subjects with early onset and long durations of hearing loss demonstrate particularly poor rate discrimination and ITD sensitivity. These latter observations apply to both human psychophysical studies (e.g., Busby et al., 1993; Ehlers et al., 2017; Laback et al., 2015; Litovsky et al., 2010) and electrophysiological studies in animals (e.g., Beitel et al., 2011; Chung et al., 2019; Hancock et al., 2010; Sunwoo, 2023; Tillein et al., 2010; Vollmer et al., 2005, 2017). Any deafness-induced structural abnormality between AN and MSO may alter the temporal accuracy and the excitatory/inhibitory balance of inputs at the MSO and may, thus, likely disrupt binaural coincidence detection and ITD discrimination (O’Neil et al., 2011; Takesian et al., 2009, for review).

#### (b) Modified by the Presence and Type of Electrical Stimulation That They Have Experienced?

The question arises whether deafness-induced deficits in rate discrimination and ITD sensitivity can be ameliorated by reinstating auditory inputs. In congenitally deaf and ND animals, chronic CI stimulation can, at least partially, restore structural abnormalities (e.g., CN: Lustig et al., 1994; O’Neil et al., 2010; Ryugo et al., 2005; MSO: Tirko and Ryugo, 2012) and functional degradations in temporal coding and ITD processing (e.g., IC: Snyder et al., 1995; Sunwoo et al., 2021; Vollmer et al., 1999, 2005, 2017a). Results from ND cats demonstrated that the effectiveness of passive stimulation on temporal coding by IC neurons depends on the temporal properties of the electric stimulation (Vollmer et al., 1999). When compared to acutely deafened adult animals, low-rate chronic stimulation (30–80 pps) failed to increase monaural temporal processing (Vollmer et al., 1999), whereas “temporally challenging” higher-rate stimulation around the maximum rate-following capacity typically found in IC neurons (∼300 pps) significantly increased the upper limit of synchronized responses to electric pulse trains, even after long durations of deafness (>3.5 years; Vollmer et al., 1999, 2005, 2017a). However, stimulation at even higher rates (≥800 pps) failed to enhance monaural temporal coding (Vollmer et al., 2017b), suggesting that enhancements in temporal processing only occur within a certain range of stimulation rates.

Moreover, experimental results imply that a critical amount of chronic electric stimulation is necessary for inducing temporal plasticity in the functionally degraded, deaf auditory system (Chung et al., 2019; Vollmer et al., 2017). In addition, behaviorally relevant stimulation is more effective than passive stimulation in driving neural temporal plasticity in the ND system, particularly in the ACx, to a lesser extent in the IC (Vollmer et al., 2017). However, the behavioral task used in the latter study did not require temporal discrimination, potentially underestimating the impact of behavioral training on temporal plasticity in the IC. Human studies support the assumption that training on temporal discrimination tasks (e.g., pitch-ranking) can enhance pulse-rate discrimination in CI subjects (Bissmeyer et al., 2020; Goldsworthy and Shannon, 2014). Note, however, that Carlyon & Deeks raise the concern that improvements in rate discrimination do not necessarily reflect sensory plasticity but could also be due to confounding factors, such as non-sensory or procedural learning.

Although stimulation- or training-induced enhancements in response precision to the TFS in monaural pathways may critically contribute to the restoration of ITD sensitivity at higher rates, recent data indicate the necessity of binaurally correlated ITD cues to restore, at least partially, the precise operation of MSO coincidence detector neurons (Sunwoo et al., 2021; Thompson et al., 2021). Whether longer stimulus durations in a behaviorally more meaningful context achieve even better outcomes in ITD sensitivity is unclear. Additionally, it remains to be tested whether or to what degree the improvements in neural ITD processing translate into enhancements in perceptual ITD discrimination.

Beyond deafness-induced degradations, technical obstacles pose challenges for CI subjects in discriminating ITDs. Conventional envelope-based CI stimulation strategies use high carrier rates (>900 pps), do not represent the TFS of the incoming signal, lack synchronization between the ears, and present envelope fluctuations insufficiently sharp to provide usable ITDs (e.g., Laback et al., 2015, for review). Chronic stimulation with binaurally uncorrelated TFS might further degrade the (potentially already impaired) ability of early-onset deaf CI subjects to effectively encode ITDs, especially at higher pulse rates (Buck et al., 2023; Rosskothen-Kuhl et al., 2021). Longitudinal measures of ITD discrimination performance starting shortly after initial CI-activation may allow this assumption to be tested.

### What If Anything Can Be Done to Improve the Temporal Processing of Localization Cues by CI Listeners?

Areas in which developments hold promise to achieve enhancements in rate discrimination and ITD sensitivity in CI listeners include (1) refined binaural signal coding strategies, and (2) enhanced recruitment of neural plasticity in the deaf auditory system. However, before discussing potential improvements, it is essential to establish fair comparisons between NH and CI listeners. As argued earlier, we believe that the observed low upper limits for rate discrimination and ITD sensitivity are not inherent limitations of electric stimulation itself. Instead, acoustic and electric stimuli yielding comparable spatiotemporal profiles of neuronal responses seem to result in similar upper limits for phase-locking and ITD sensitivity. Future studies comparing responses of neurons in the IC, and desirably in the MSO and LSO, to transient acoustic stimuli (chirps, clicks) and electric pulses across wide ranges of pulse rates can help validate this assumption.

Although the exact mechanisms linking hyper-synchronicity in response to electric stimulation to the low limits in rate discrimination and ITD sensitivity are still unknown, better controlling the spatiotemporal dispersion of electric responses to approximate temporally dispersed response patterns to TFS in acoustic stimuli may enhance rate discrimination and ITD sensitivity. For instance, refined designs of pulse shape, such as on-ramped pulses, can enhance selectivity along the tonotopic axis (e.g., Ballestero et al., 2015) and may evoke responses with increased jitter, more closely resembling response patterns to temporally dispersed acoustic stimuli. The increase in synaptic jitter which is expected to reduce the artificial hypersynchronicity of CN and MNTB responses may impact binaural input integration at the MSO and LSO to the effect of improved ITD sensitivity in CI users at higher rates (Müller et al., 2023; Myoga et al., 2014). Further research on how binaurally correlated or uncorrelated jittered inputs at a wide range of mean pulse rates affect electric ITD processing in MSO and LSO would be valuable.

Beyond mechanisms related to pulse design, the temporal structure of the pulse train itself is a potential target for improving ITD sensitivity. Introducing binaurally coherent jitter in electric pulse trains has shown promise in increasing ITD discrimination at high pulse rates (Laback and Majdak, 2008). Hancock et al. (2012) provided evidence that this effect is mainly due to irregularly occurring short interpulse intervals. Thus, in addition to jittering, the insertion of interspersed short interpulse intervals in an otherwise periodic pulse train offers an alternative strategy for pulse train modification to improve ITD sensitivity without compromising speech intelligibility (Buechel et al., 2018; Srinivasan et al., 2018, 2020).

We contend that deafness-induced degradations in the upper limit of rate discrimination and ITD sensitivity represent a permanent “hard-wired” upper limit for temporal processing. Within a limited range of “temporally challenging” pulse rates (∼300–600 pps), especially when combined with behavioral training on temporal discrimination tasks, chronic electric stimulation can recruit temporal plasticity in the deaf central auditory pathway, restoring aspects of neural temporal processing (e.g., phase-locking, latency, jitter; Vollmer et al., 1999, 2005), all of which may contribute to enhanced rate discrimination and ITD sensitivity at higher rates. The effective rates for temporal plasticity appear to align closely with the typical upper limit of electric pulse rates for extracting ITD information (∼300 pps). This is, however, well below the high stimulation rates (>900 pps) used in conventional speech processing strategies. Thus, optimizing processing strategies to deliver binaurally correlated ITD cues at such lower “temporally challenging” TFS rates while, at the same time, providing sufficient envelope sampling to maintain speech intelligibility remains a viable challenge.

We propose that behavioral training on temporal discrimination tasks, possibly facilitated through feedback-controlled at-home training sessions, could be highly effective in engaging plasticity mechanisms for targeting temporal processing capabilities in CI users. To assess stimulus properties *and* training strategies that most effectively enhance rate discrimination and ITD sensitivity in the deaf auditory system, longitudinal studies with chronic neural recordings from the same neurons in awake preparations, along with measures of behavioral performance, including appropriate controls to separate unspecific training effects from specific mechanisms of auditory neural plasticity, are essential.

## Andrej Kral and Jochen Tillein

### To What Extent Are the Limits on CI Users’ Use of Purely Temporal Cues to Perceive the Pitch and Spatial Location of Sounds

#### (a) Due to a Fundamental Biological Limitation?

#### (b) Modified by the Presence and Type of Electrical Stimulation That They Have Experienced?

Neural phase-locking to the stimulus represents an important cue for perception. However, temporal information is not independent of spectral information, for example, in pitch perception (Oxenham, 2018; Oxenham et al., 2004) or binaural cues that are integrated with level and monaural spectral cues (Keating & King, 2013). This suggests that temporal cues cannot be considered in separation from spectral cues, and that integration of both is critical for auditory performance. Also, intensity effects interact with temporal (and place) coding.

The highest temporal acuity is observed in AN fibers with some improvements through coincidence detection in the bushy cells of the CN (Joris et al., 1994). From the CN to the ACx, the sensitivity to temporal structure of the stimulus, as for example, measured by phase-locking to the stimulus, decreases (Eggermont, 2001).

In CI subjects, temporal information may be affected at three levels:

1. In the signal (speech) processor itself. The constant stimulation rate of ∼1500 pps does not transmit envelope temporal information beyond ∼350 Hz, since four datapoints per period are required to sufficiently represent the signal (McKay et al., 1994). Furthermore, faint portions of the spectrum are eliminated in CIs due to the narrow dynamic range of electric stimulation and the coding strategy. These portions may provide important temporal information in complex sounds (Carney, 2024; Mao & Carney, 2015). Since the processors of both ears are not synchronized to each other, it is difficult to provide consistent binaural timing information at µs precision within longer time windows (Culling & Colburn, 2000; Kolarik & Culling, 2009). High-frequency temporal information is thus sufficiently represented neither in the monaural nor in the binaural domain in current-day CI processors. Finally, presenting temporal information by amplitude modulation of a pulse train of constant rate, as in CIs, is less efficient and robust compared to varying stimulation rate, further blurring the pitch percept even at lower frequencies (Goldsworthy et al., 2021).
2. At the electrode–tissue interface (in the cochlea) temporal information itself is not degraded. Recordings from the AN document a high level of phase-locking using electrical stimulation (Dynes & Delgutte, 1992; Hartmann et al., 1984; Shepherd & Javel, 1997; Tillein et al., 2015). Electrical phase-locking exceeds the frequency limit for acoustic phase-locking (near 4 kHz) and shows superior phase-locking both below and above the limit. However, dynamic range is compressed to a few dB with electric stimulation (Hartmann et al., 1984; Shepherd & Javel, 1997), quickly saturating responses in the temporal domain. While differences in thresholds of individual fibers can expand this in population to ∼20 dB (Sato et al., 2016), this is much less than in normal hearing animals. Taken together, temporal information in the AN is more regular and phase-locking more precise with electric than acoustic stimulation, leading to *hypersynchronization* of neuronal activity (Sato et al., 2017). Place (spectral) information, on the other hand, is substantially degraded with monopolar stimulation (Bierer & Middlebrooks, 2004; George et al., 2015; Kral et al., 1998; Snyder et al., 2004). Degraded spectral information at high frequencies smears higher-order harmonics that fall into one single stimulation channel and become unresolved. This is critical for complex pitch perception.
3. In the central auditory pathway (in the brain). In animals with no previous period of hearing loss, temporal representation was largely comparable between acoustic and electric (CI) stimulation. Modulation transfer functions determined in the auditory midbrain revealed similar cut-off frequencies with electric and acoustic stimulation (Shepherd et al., 1999; Snyder et al., 1991; Vollmer et al., 1999). The ability to resolve ITDs down to < 100 µs has been confirmed with electric stimulation, very similar to acoustic stimulation, in cats (Smith & Delgutte, 2008; Smith & Delgutte, 2007; Tillein et al., 2010) and other species (Buck et al., 2021; Chung et al., 2014, 2019). In the brainstem, the ITD processing relies on bushy cells that receive convergent input from 2 to 4 fibers from neighboring locations of the AN and by coincidence detection improves the temporal timing in the AN (Joris et al., 1994). With CIs these receive a strong drive due to highly synchronous stimulation of spiral ganglion cells. Bushy cells can be effectively stimulated with CIs in the rat (Paolini & Clark, 1998), consistent with high fidelity of extraction of ITD information. For extracting the envelope of the sensory input, stellate cells are key: they integrate smaller (neighboring) portions of the AN and code the envelope (Cao et al., 2019; Doucet & Ryugo, 1997; Schofield et al., 2014). Intracellular recording confirmed that stellate cells can also be effectively stimulated with CIs (Paolini & Clark, 1998). However, pauser (octopus) cells that compensate cochlear traveling delays (Cant & Benson, 2003; Golding et al., 1999) are probably only weakly active when the cochlea is stimulated electrically (i.e., with no cochlear delays). They are critical for detecting auditory transients across the cochlea (same sound onsets at different cochlear locations). Nonetheless, so far deficits of temporal processing in acutely deafened and CI-stimulated animals have not been consistently observed.

Overall, the physiology suggests limited issues with temporal envelope and ITD processing in electric stimulation and no substantial loss of timing information by neural processing itself. Despite these physiologic observations, there is a fundamental temporal deficit observed in human psychophysics, even in direct stimulation bypassing the speech processors: while normal hearing listeners are sensitive to temporal pitch rates >700 Hz even when stimulated using unresolved harmonic complexes (Carlyon & Deeks, 2002) or even >1500 Hz using TFS (Verschooten et al., 2019), pitch perception in the cochlear-implanted subjects saturates near 300–400 Hz and temporal pitch discrimination is degraded beyond that limit (Carlyon et al., 2008; Shannon, 1983; Zeng, 2002; see Figure 2). In contrast to hearing subjects, bilateral CI subjects rely heavily on ILD cues in spatial localization (Seeber & Fastl, 2008) and their sensitivity to ITDs is compromised for high stimulation rates even in direct stimulation bypassing the CI processor (>100 pps; van Hoesel, 2007; van Hoesel et al., 2009; Figure 3). Does this suggest a general issue with timing in electric hearing that has been missed in physiology that particularly relates to higher-frequency temporal information, even if bypassing the processor? We will elaborate this question from three perspectives.

##### Plasticity and Development?

One complication is brain plasticity that changes the representation of the stimulus depending on the type of stimulation provided or its absence (review in Kral et al., 2019; Kral & Tillein, 2006). In place coding degenerative processes and plasticity with CIs have been observed developmentally (Fallon et al., 2009; Klinke et al., 1999; Kral et al., 2002; Raggio & Schreiner, 1999). Central temporal precision also undergoes degradation in deafness (Middlebrooks, 2018). After chronic CI stimulation during development, plasticity in the temporal representation has been demonstrated in the IC (Shepherd et al., 1999; Snyder et al., 1991; Thompson et al., 2021; Vollmer et al., 1999) and the ACx (Vollmer & Beitel, 2011). Similarly, pitch perception changes over time in human CI users (Reiss et al., 2007). Additionally, congenital deafness degrades sensitivity for ITDs both in animals (Hancock et al., 2010; Tillein et al., 2010, 2016) as well as in humans (Gordon & Kral, 2019; Litovsky, 2011; Litovsky et al., 2010). Thus, for developing the exquisite sensitivity for binaural timing cues, hearing experience in childhood is essential. This all suggests that developmental hearing loss, but potentially also absence of the appropriate (consistent!) CI stimulation during development, may degrade temporal processing. Nonetheless, the discrepancy between physiology of temporal processing and psychophysical deficits in temporal domain of adult-deaf CI subjects cannot be reconciled by effects of developmental plasticity.

##### Apical Cochlear Fibers Are Special?

Temporal information needs to be provided consistently with place information in CIs, too (Rader et al., 2016; Schatzer et al., 2014). Different characteristics of neurons in the auditory brainstem suggest that the apical spiral ganglion cells may additionally provide more precise temporal information compared to the more basal ones. In bushy cells projecting to the trapezoid body, higher precision of phase-locking was observed in fibers with characteristic frequencies <1 kHz than those above 2 kHz (Joris et al., 1994). CIs stimulate preferentially the basal cochlea and might not reach these fibers. Indeed, intraneural and cochlear apex electric stimulation provided a better phase-locking than conventional CIs, suggesting a “specialized apical temporal processing pathway” (Middlebrooks & Snyder, 2010). Some improvements have been obtained by implementation of TFS in the most apical electrodes of long electrode carriers, which are likely to approach the end of the second turn of the cochlea (Lorens et al., 2010; Müller et al., 2012; Riss et al., 2011, 2014, 2016; Vermeire et al., 2010), but the outcomes were not consistent with a complete resolution of the temporal issue by apical fine-structure stimulation only.

##### Issue in Temporal Volley Coding

We think that the discrepancy between psychophysics and physiology is related to the different level of study: while psychophysics integrates all information available and accessible by the brain, physiology is limited—particularly when studying temporal properties—to individual or few neurons. In physiology, the whole excitation profile within a structure can be recorded at the same time using multielectrode arrays (Bierer & Middlebrooks, 2004; Sato et al., 2016). These are, however, not useful for recording the single fibers of the AN. There is still insufficient insight into the representation of the electric stimulation in the whole excitation profile on the trial-by-trial basis. When analyzing the modulation transfer functions in the IC with electric stimulation, studies consistently report the cut-off frequencies of only ∼300–400 Hz (Middlebrooks & Snyder, 2010; Vollmer et al., 2017a) and only few individual midbrain neurons show modulation at higher frequencies with acoustic stimulation (Langner & Schreiner, 1988). It is at present unclear whether these few neurons are indeed of functional relevance or whether the temporal code has already been largely transformed to a place code in the IC. Here again we lack understanding of the temporal representation. This requires more studies, particularly those focused on the AN.

Due to refractoriness, single AN fibers’ firing rates saturate at 300–400 Hz. For higher-frequency periodic stimuli, the neurons respond in a phase-locked manner but do so in different fibers in different periods (in every second or third, stochastically). Pooling (integrating) fibers throughout the large portion of the excitation profile (characteristic frequencies) allows extraction of temporal structure for periodicities >300 Hz (volley coding).

There is a principal issue with this coding in electric stimulation: there is both an abnormally high spread of excitation throughout the cochlea and an abnormally high synchrony that together yield activity between different AN fibers highly correlated over large portion of the nerve. In the most extreme case if all activated fibers responded to the same period of a periodic high-frequency stimulus, responsiveness would be limited in the subsequent period due to refractoriness, and this would be similarly true in all active fibers. Excitation would be possible only on the third or fourth period, again in (nearly) all fibers in the same period. Volley coding thus would not provide sufficient variety of phase-locking information in different fibers and lacks complementarity in different periods of the stimulus. In the absence of the physiological variability of this information, frequency representation in the temporal code is limited to the single-fiber limit of 300–400 Hz. Since synchronization further increases with increasing stimulation level, the situation is aggravated by the small dynamic range of single fibers (∼3 dB for pulsatile stimulation, Hartmann et al., 1984; Javel & Viemeister, 2000, only ∼20 dB in the population, Bierer & Middlebrooks, 2004; Sato et al., 2016). Such *hypersynchrony* of the auditory responses to electric stimulation combined with small dynamic range thus heavily compromises transmission of complementary information in the volley (population) code. This is consistent with all the above results on human CI recipients and also with the good ITD sensitivity observed at low pulse rates but degrading at higher pulse rates when they exceed the phase-locking limit of individual nerve fibers.

Degeneration of spiral ganglion cells further reduces the temporal variability across different fibers by reducing their number (Zhou et al., 2019). This further compromises temporal information and is probably one reason for the high variability of psychophysical outcomes between subjects, for example, in ITD sensitivity (van Hoesel et al., 2009). In binaural stimulation, binaural electrode matching is critical (Kan et al., 2015). Loss of AN fibers, particularly patchy one, thus complicates binaural integration in time domain, but also affects temporal pitch processing (Zhou et al., 2019).

### What Would Change Your Mind?

There are several approaches that could falsify our claim of increased between-fiber synchrony. Some authors point to inconsistencies between their loudness models and compound action potential data and suggest that the electric stimulation effects on saturation are not as large as assumed (McKay et al., 2013). Use of multielectrode recording in the AN, allowing to analyze this together with spiking synchrony between fibers, would eventually provide data on this issue. A less direct possibility is the recording in the CN using multielectrode arrays; here the difficulty is to record from functionally homologous neurons, given that CN includes a high diversity of neurons. Psychophysically, use of focused stimulation (either by current focusing or by modiolar-hugging electrodes) that provides significantly reduced channel interaction should also have a byproduct of slightly increased temporal limit, provided the same signal is fed to neighboring channels asynchronously in TFS. However, the issue of synchronized activity in all fibers within a given channel remains and therefore the effect size in such experiment could be rather small. Use of simultaneous analog stimulation strategies that provided good speech perception in modiolar-hugging electrodes (e.g., Battmer et al., 1999) might shed light on whether the limit is given by the stimulation configurations used in the present CI designs.

### What If Anything Can Be Done to Improve the Temporal Processing of Pitch and Localization Cues by CI Listeners?

Several limitations result directly from current speech processing and stimulation strategies. To improve the localization ability with CIs we need synchronized binaural processors. We have to put further effort into new strategies that provide more fine-structure information at the correct place, and more focused stimulation strategies or electrodes closer to the modiolus to provide better channel separation at the cochlear base (Quass & Kral, 2024). Cochlear anatomy-based surgery and fitting, based on precise anatomical models that account for interindividual variability of the human cochlea (Avci et al., 2014; Pietsch et al., 2022; Schurzig et al., 2023), is the first step toward an individualized cochlear implantation. Current-focusing approaches could be beneficial in providing more place-structured input to the cochlea particularly at high sound pressure levels where faint percepts are not an issue. However, several of the above suggestions require a hardware redesign of the processors and new procedures for processor adjustments, thus substantial investments. Measures to identify damage to cochlear nerve, particularly the patchy form of damage (Arenberg Bierer, 2010; Konerding et al., 2022; Ramekers et al., 2014), may allow differentiation of those patients who may not profit from focused strategies. However, the fundamental issue with volley coding is difficult to resolve with any artificial stimulation of spiral ganglion cells without restoring a functional hair cell synapse. Here, the optimal use of any functional hair cells and their preservation during implantation is the best approach today. Hair cells regeneration remains an elusive future goal.

## Jan Schnupp and Nicole Rosskothen-Kuhl

### To What Extent are the Limits on CI Users’ Use of Purely Temporal Cues to Perceive the Pitch or Spatial Location of Sounds Due to

#### (a) A Fundamental Biological Limitation?

**Figure 4.**
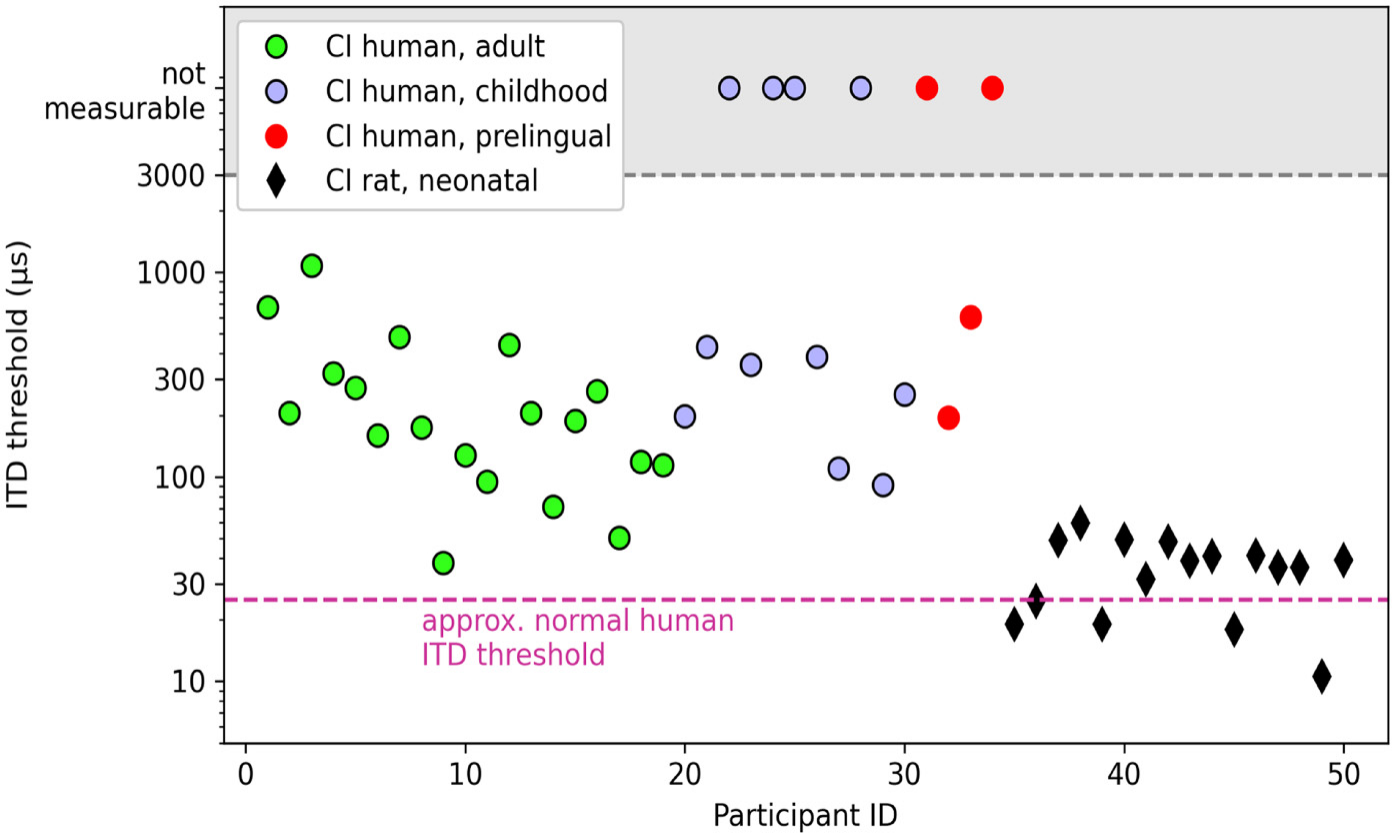
Comparing behavioral discrimination thresholds for electric pulse timing ITDs observed in 34 human biCI patients who lost their hearing either in adulthood (green circles), childhood (blue circles), or pre-lingually (red circles), along with thresholds of 16 neonatally deafened, adult biCI supplied rats (black diamonds). Electric pulse train stimuli were delivered to electrodes in the middle turn of the cochlea of each ear, using experimental processors that allow the delivery of precise pulse timing ITDs. Human data from Litovsky et al. (2012). Rat data from Buck et al. (2023). biCI = bilateral cochlear implant; CI = cochlear implant; ITD = interaural time difference.

Here we will concentrate on the use of ITD cues by bilateral cochlear implant (biCI) patients, leaving consideration of pitch to our colleagues, and we interpret the question to mean: “Are biological limitations to blame for the typically poor sensitivity of biCI patients to ITDs?.” It is well known that the NH auditory system can process ITDs as brief as ∼20 μs to localize sound (Brown & May, 2006; Klumpp & Eady, 1956) and analyze auditory scenes (Klump, 2006). However, ITD sensitivity is generally impaired in biCI patients, even when they are tested with experimental processors to deliver precise pulse timing ITDs (Figure 3). Furthermore, ITD sensitivity is often particularly poor in patients with early hearing loss and thus only limited experience of acoustic hearing. This is illustrated, for example, by Litovsky et al. (2012), who reviewed data from 34 biCI patients with different onset of deafness. We replotted their data in Figure 4. The circles show ITD thresholds of biCI patients who lost their hearing either in adulthood (green circles), during childhood (blue circles), or prelingually (red circles). For comparison, the dotted purple line shows the approximate ITD threshold of NH humans (Brughera et al., 2013; Klumpp & Eady, 1956). The adult deaf CI patients shown in Figure 4 have a mean ITD threshold of ∼270 μs, which means that the spatial resolution afforded by this cue is on average *ten times* worse than that of NH participants. (The logarithmic *y*-axis may make the deficit appear smaller than it actually is. For small ITDs near the midline, ITD scales approximately linearly, not logarithmically, with azimuthal sound source location.) Figure 4 also makes it easy to appreciate that patients who became deaf as children or babies have a fairly high risk of exhibiting ITD sensitivity so poor that no thresholds can be measured at all. While Thakkar et al. (2020) did not find a clear effect of age at onset of deafness, the study of Ehlers et al. (2017) confirmed that prelingually deaf patients exhibit particularly poor ITD sensitivity with thresholds too large to measure in seven out of 10 patients.

The especially poor ITD sensitivity of early deaf patients has led to the suggestion that early deafness may hinder the proper development of binaural timing circuitry in the auditory brainstem suggesting a “critical period hypothesis” (Ehlers et al., 2017; Kral, 2013; Kral & Sharma, 2012; Litovsky et al., 2012). However, while the human data in Figure 4 indicate that early binaural experience influences ITD sensitivity, it cannot be the only factor for the poor sensitivity, as adult-deafened patients also show elevated ITD thresholds (Cleary et al., 2022; Laback et al., 2007; Litovsky et al., 2012; Majdak et al., 2006; Thakkar et al., 2020).

Unfortunately, ethical and technical limitations on research with human patients make it extremely difficult to identify and isolate the different factors responsible for these poor outcomes. Most or all of the electrode channels of all currently available clinical processors only deliver accurate pulse timing cues in rare experimental settings. Patients are therefore given little to no opportunity to become skillful in utilizing pulse timing cues. Rather, clinical practice requires that any CI participant will have invested countless hours of practice in trying to make the most out of the quite unnatural input provided by their devices (Tyler et al., 1997) before they sign up for a study. Their clinical need for copious exposure to input that has been stripped of informative pulse timing cues makes it effectively impossible to set up experiments that can shed a clear light on what a CI-stimulated auditory pathway might be capable of if it was given consistent access to input optimized for temporal coding.

To work around this major confound, we have developed a behavioral animal model that allows us to study CI pulse timing ITD sensitivity while fully controlling our animals’ acoustic or electric hearing experience. We first demonstrated that NH rats are easy to train in ITD lateralization tasks using acoustical stimuli and capable of discriminating ITDs of ∼50 μs (Li et al., 2019). We then tested the ability of ND rats which were bilaterally implanted in young adulthood to lateralize binaural electrical pulse trains based on ITD (Buck et al., 2023; Rosskothen-Kuhl et al., 2021). All animals thus underwent a phase of severe-to-profound hearing loss before bilateral implantation in young adulthood. The black diamonds in Figure 4 show behavioral ITD discrimination thresholds from 16 ND biCI rats tested with 300 pps pulse trains with a remarkably low mean threshold of only 35 µs (Buck et al., 2023), which is comparable to the thresholds for NH humans and rats (sign-rank test against comparable data from Li et al. [2019], *p* = .16). The mean ITD threshold for these ND biCI rats is thus almost *eight times* better than that of the adult deafened human patients (Figure 4). Not a single one of these biCI animals had elevated ITD thresholds, despite the fact that they were severely deprived of auditory input throughout their development up to sexual maturity.

Our rat data may appear particularly surprising in the light of earlier reports that congenitally deafened cats (Hancock et al., 2012, 2013; Tillein et al., 2010, 2016) and ND rabbits (Chung et al., 2014, 2019) exhibited comparatively poor ITD tuning of auditory midbrain or cortex neurons under CI stimulation. However, these earlier studies sampled ITDs in rather large steps, and tested only very few ITD values within the physiological range of the animals, limiting the usefulness of these datasets for predicting an animal's likely ability to discriminate ITDs as small as a few tens of μs. We therefore sampled ITD tuning curves of IC multiunits in freshly implanted ND biCI rats in small, 20 μs steps, focusing on the animals’ physiological range (Buck et al., 2021). Even in these completely inexperienced and developmentally deprived animals, 85% of multiunits showed at least some tuning to ITDs in the ±160 μs range, and many multiunits modulated their firing rates substantially in response to ITD changes of only a few tens of μs. In that study, we further performed an analysis demonstrating that coarser sampling of ITDs over a range exceeding that naturally experienced by the animal led to a substantial reduction in the number of ITD-sensitive IC neurons from 85% to 53% and was similar to the observations of Hancock et al. (2010, 2013) and Chung et al. (2019) in congenitally or early-deafened animals.

These results suggest that there may be no compelling biological factors preventing the CI-stimulated auditory pathway from exhibiting near normal ITD sensitivity, provided that the temporal information can be delivered appropriately. Technical factors are likely to be more relevant here, and two of those we have been able to examine in our rat models, namely (1) pulse rate, and (2) the relative effectiveness of envelope and pulse timing as carriers of temporal information.

Pulse rate matters because, at high rates, the auditory pathway is no longer able to resolve individual pulses (Hancock et al., 2017), and the ability to use pulse timing should decline when pulses are not well resolved. However, we recently showed that biCI rats can lateralize small ITDs even at pulse rates as high as 900 pps. Our current working hypothesis is therefore that their auditory systems accurately encode the onset of bursts of pulses, although we have obtained preliminary evidence that pulses beyond the first can contribute (Rosskothen-Kuhl et al., 2024). The fact that clinical devices need to operate at fairly high pulse rates in order to adequately sample the envelopes of important sounds such as speech therefore need not become an obstacle to good ITD sensitivity in hearing with biCIs.

However, if our animals really use the pulse train onset rather than the TFS of a pulse train to lateralize high-pulse-rate stimuli, does that mean that they are processing “envelope ITDs”? If so, our emphasis so far on pulse timing rather than pulse train envelope timing as the carrier of timing information could be misguided. Previous studies on patients have investigated the relative effectiveness of pulse versus envelope timing ITDs (Majdak et al., 2006; Noel & Eddington, 2013; van Hoesel & Tyler, 2003), but given the limitations faced by human studies referred to above, these studies could only use patients who had become adapted to clinical processors, and who required relatively large ITDs and low pulse rates to be able to perform the required tasks. With our biCI rat model, we were able to investigate the relative effectiveness of pulse and envelope timing ITDs for high pulse rate (900 and 4500 pps) stimuli, using ITD values (+80 μs) that normal listeners can easily lateralize, but that are beyond the capability of most biCI patients. Our results showed unambiguously that ND biCI rats were many times more sensitive to pulse timing than to envelope ITDs, irrespective of envelope shape and pulse rate (Schnupp et al., 2023).

How can pulse timing drive ITD discrimination even at such high pulse rates? As stated above, our current hypothesis is that, at high pulse rates, the auditory pathway produces mainly onset responses driven by the first supra-threshold pulse in a burst of pulses. Recordings by Hancock et al. (2017) support that idea. If these onset responses align with the temporal grid established by pulse timing generators in each CI processor, then pulse timing strongly influences temporal processing even at pulse rates so high that individual pulses cannot be resolved. In this respect, an auditory system stimulated by pulsatile CI stimuli differs importantly from an acoustically stimulated one. Normally, the physiology of inner hair cells applies a low-pass filter to high-frequency fine-structure information, but no such filtering step occurs prior to the AN of CI patients. Consequently, many of the classic distinctions between envelope and fine-structure ITD discrimination that have emerged in the acoustic binaural hearing literature are not directly transferable to the CI case.

We have seen that the auditory pathway is intrinsically exquisitely sensitive to the timing of electric pulses (and much less so to the timing of pulse train envelope features), but if this is so, then why do typical biCI patients nevertheless struggle to lateralize pulse timing ITDs? We do not believe that it is currently possible to provide a definitive answer to this, but most likely a large part of the problem is that current clinical devices give CI patients so little opportunity to hone their ability to process sub-millisecond temporal cues.

#### (b) Modified by the Presence and Type of Electrical Stimulation That They Have Experienced?

A key issue that is easily forgotten is that, for an auditory pathway that has evolved to use its exquisite sensitivity to temporal cues to solve sophisticated pitch and spatial discrimination tasks, being bombarded with entirely uninformative pulse timing intervals may be worse than useless, it could be disruptive. Normally, ITD cues are subconsciously combined with ILDs and other cues to form an integrated percept of source location. When ITD and ILD cues contradict each other, the auditory system normally tries to compute a compromise location estimate. A sound that is louder in the left, but slightly earlier in the right ear may thus be perceived near the midline, a phenomenon referred to as “time-intensity trading.” The relative strength of ITD and ILD cues in shaping the overall spatial percept is quantified by the “time-intensity trading ratio” (TITR) in μs/dB (Joris et al., 2008; Trahiotis & Kappauf, 1978). Clearly, it would be of great interest to know TITRs for the CI-stimulated auditory system in its native state. The more TITRs favor ITDs (the smaller the TITR values), the greater the potential for the uninformative pulse timing ITDs to confound whatever useful ILD information a CI patient may receive.

As-yet unpublished data recently collected in experiments on our ND biCI rats indicate that TITRs in the native, CI-stimulated auditory pathway are no larger than 20 μs/dB. What would comparatively small TITRs imply for a prelingually deaf biCI patient when they first experience bilateral clinical CI stimulation? Many clinical processors deliver pulses at fixed rates close to 1000 pps, and with independent pulse train generators in the left and right ears drifting in and out of phase, this would imply that the pulse timing ITDs they receive are random numbers drawn from an interval of ±500 μs (see Figure 3 for an illustration of competing pulse and envelope ITDs). By simple extrapolation of a TITR of ∼20 μs/dB, one would predict random pulse timing ITDs as large as 500 μs to be able to confound informative current amplitude ILDs as large as 25 dB. To put this number into perspective, we have to remember that the total dynamic range of usable CI pulse amplitudes is often no larger than ∼10–20 dB, and CI patients are known to resolve fewer stimulus intensity steps than NH listeners (Zeng et al., 2002). Random pulse timing ITDs would then be able to confound even the largest ILDs that can possibly be delivered. Admittedly, the simple linear extrapolation we made here may not be entirely valid, but our new results suggest that “wrong” pulse timing ITDs of even modest size have the potential of confounding sizable “correct” ILDs. BiCI patients must therefore probably become insensitive to the nonsense pulse timing ITDs that they are constantly bombarded with if they are to be able to derive any binaural benefits at all from their clinical devices, which would explain the very poor ITD sensitivity seen in many CI users. This process appears maladaptive in that it blunts what would normally be a delicate sensory faculty, but it is adaptive in a context where paying attention to pulse timing has nothing to offer but confusion. These ideas that maladaptive plasticity in response to inappropriate timing pulses may play an important role are testable in animal experiments, and corresponding studies are currently under way in our laboratories.

### What Would Change our Minds?

Based on our animal research we attribute the currently typically poor ITD sensitivity of most bilateral CI patients to inadequate stimulation provided by suboptimal technology. CI patients with both ANs and the auditory pathway in good condition, and good electrode–nerve interfaces should be able to achieve ITD discrimination thresholds no worse than seen in NH listeners if given consistently accurate ITDs delivered via pulse timing rather than pulse envelope. We would like to emphasize once again that limited exposure to informative pulse timing ITDs only during relatively brief testing sessions is not sufficient. What would change our mind would be evidence that our rats are unrepresentative because they are somehow biologically better at ITDs than humans. However, this seems very unlikely, as most researchers assumed the opposite until recently. Finally, we would like to propose an experiment in early-deafened bilateral CI rats that receive only uninformative pulse ITDs from the onset of stimulation to confirm or refute our hypothesis of the importance of informative pulse timing ITDs. If the animals do not show poorer ITD sensitivity as a result, this could change our mind.

## What If Anything Can Be Done to Improve the Temporal Processing of Pitch and Localization Cues by CI Listeners?

Our results suggest that poor binaural temporal processing in CI hearing is by no means inevitable, and that in many cases it may be acquired as the auditory pathway adapts to a form of electrical stimulation which provides little or nothing in the way of usable sub-millisecond temporal cues. On that basis, it ought to be possible to improve temporal processing in many CI patients simply by making sure that they receive a “sensory diet” which is “enriched” in appropriate pulse timing cues. The fascinating observations by Goldsworthy and Shannon (2014) that CI patients can learn to improve their pulse-rate discrimination ability through practice also support this point of view. We do, however, appreciate that, firstly giving patients better input is easier said than done, and secondly, that human patients are highly diverse, and our model is not equally applicable to all of them. Our recent animal work is encouraging, but it has so far set aside the problem that real patients need informative pulse timing delivered alongside effective cochlear place coding of speech formant information across multiple electrode channels. The CIS-derived strategies running in current clinical processors sacrifice TFS information in order to make it easy to reduce channel interactions that might impair place coding. To our minds there are no good reasons to believe that the trade-offs made in these design decisions are anywhere near optimal, but it remains unknown how one would design a strategy which jointly optimizes place and temporal information. Solving this formidable problem will require much more sophisticated, coordinated studies than we have seen so far, but we are confident that we will be able to do this collaboratively in the future to improve spatial hearing of CI patients.

## Summary and Discussion

The questions posed in the Introduction led to consensus on some issues and to controversy on others. This section presents an overview and discussion of some of the main points. To aid the reader Table 2 provides a bullet-point summary of each contributor's main arguments.

**Table 2.** Summary of Viewpoints on the Neural Basis for Limits on Temporal Processing and of Their Possible Modification by Auditory Experience and Training.

**Carlyon and Deeks**
Temporal pitch perception varies across electrodes within the same listener as well as between listeners. The between-listener differences could in principle be due to plasticity or experience, but this is unlikely to explain the (sometimes substantial) across-electrode within-listener differences. Even when the pitch of a pulse train increases up to some value, the actual pitch perceived may be lower than this upper limit. There is no convincing evidence from training studies that it is possible to overcome the “upper limit,” even though, as with most tasks, performance generally improves overall with practice. There is no evidence that between-listener differences in the upper limit or rate discrimination correlate with age or duration of deafness. Temporal pitch perception for some listener/electrode combinations is similar to that when analogous stimuli (filtered pulse trains) are presented to NH listeners, at least at low rates. It is unlikely that any amount of experience/plasticity will lead to an improvement over the (mediocre) performance obtained by NH listeners with those stimuli. *Pitch perception may be improved by presenting the same TFS or F0 information to multiple apical channels, or by future technologies that more selectively excite neurons innervating the cochlear apex*.
**Goldsworthy**
Pitch perception and sound localization are supported by temporal processing mechanisms exquisitely expressed in auditory physiology. These mechanisms degrade with deprivation, but some recovery occurs after sensory restoration. Presently, cochlear implants do not encode acoustic temporal fine structure into electrical stimulation; consequently, the extent that restoration of timing cues will improve pitch and sound localization is unknown. Electrode psychophysics provides an important middle way to couple precise stimulation with active listening exercises for pitch and sound localization. Discrimination of temporal cues for pitch and sound localization improves with training, but the extent that learning continues with long-term exposure is unknown. *Future studies should combine temporally precise stimulation with long-term training of pitch and sound localization. Doing so will determine the extent that cochlear implant outcomes are limited by physiology or by existing stimulation strategies*.
**Litovsky**
Bilateral CIs are limited by several factors, including lack of obligatory coordination or synchronization of inputs to the two ears. A related issue is the actual limited encoding of binaural cues. Synchronized stimulation using research processors reveals enormous between-listener variability in sensitivity to ITDs that can be attributed to age- and experience-related factors. It is better in people who have received normal acoustic input during development and in those with shorter periods of binaural auditory deprivation. If children grow up with bilateral CIs that fail to deliver low-rate, synchronized and well-preserved ITDs, their auditory system may lose the capacity to process ITD cues even when provided by future processing strategies that preserve those cues. Neural health at individual electrode sites may be critical, and monaural rate sensitivity and binaural sensitivity for ITDs may be limited by a shared mechanism. ITD-preserving strategies could provide binaural hearing comparable to NH when provided in early childhood or to adults with normally developed binaural processing prior to deafness. *One potential strategy could convey ITDs in the timing of low-rate electrical pulses on some electrodes while accurately encoding the envelope with high-rate pulse trains on other electrodes*.
**Delgutte and Chung**
The perceptual limitations on rate pitch and binaural processing with CIs are not caused by a lack of precise temporal information in the auditory nerve. However, the abnormally broad spatio-temporal pattern of activation and the excessive across-neuron synchrony may impair the ability of central inhibitory and suppressive circuits to process the available temporal information. Deficits in the perception of temporal and binaural cues in CI users are due to fundamental neural limitations. In the auditory midbrain and cortex, both neural ITD sensitivity and the coding of temporal pitch are degraded at higher electrical pulse rates, consistent with perceptual limits in CI users. Deficits in the neural representation of pitch are greatest for the rate code, which has received relatively little experimental attention despite its likely importance for pitch perception at higher frequencies. Neural ITD sensitivity to bilateral CI stimulation is further degraded in animals that experienced auditory deprivation during development. This degradation can be partially reversed by providing meaningful ITD cues through bilateral CIs during maturation. *Improvements could be obtained using technologies that achieve more-selective and less-synchronized patterns of stimulation, or, if the plasticity effects observed in neural recordings prove to have behavioral consequences, by more-immersive training protocols*.
**Vollmer and Ohl**
Designing experiments allowing inference on neuronal processing of “purely temporal” cues poses challenges, because changes in the temporal properties of a stimulus affect both its temporal and spectral characteristics. In the auditory midbrain, limitations in rate discrimination and ITD sensitivity to electric stimulation are consistent with perceptual limits in CI subjects. These limitations may be attributable to the broad and highly synchronized spatiotemporal activation patterns in response to electric pulse trains. The actual amount and time scale by which the current upper limits can be pushed by improving stimulation strategies and pulse designs are underexplored, as is the degree to which changes in neural observables translate into changes in perception. Neural phase-locking and ITD sensitivity are degraded by auditory deprivation. Chronic electric stimulation within a limited range of “temporally challenging” pulse rates and providing binaurally correlated ITD cues may partially restore this degradation, especially when combined with behavioral training on temporal discrimination tasks. *Designs of individual pulses or of entire pulse trains that more closely resemble temporally dispersed and more selective response patterns to acoustic stimuli may enhance rate discrimination and ITD sensitivity*.
**Kral and Tillein**
Temporal processing in cochlear implants is limited by hypersynchrony between different auditory nerve fibers, limiting the temporal information available in volley (population) coding. This leads to limitations in temporal pitch processing as well as in processing of the most important binaural cue, the interaural time difference. This aspect is difficult to study in physiological experiments since it requires multielectrode recordings from the auditory nerve fibers. This leads to a discrepancy in physiological and psychophysical findings. Temporal processing of electric stimulation is further aggravated by the large current spread with monopolar configuration, compressed electric dynamic range, and by degeneration of auditory nerve fibers. Central processing additionally contributes in cases of early hearing loss, where sensory experience is required for establishing and maintaining appropriate representation of binaural cues. *More precise information about the cochlear health status might help to adapt electrical stimulation to the individual cochlear hardware. Current focusing may improve the issue, but will not eliminate it. Use of longer electrodes and implementation of temporal fine structure at the apical contacts may help, but more extensive changes in the design of the implant hardware may be required*.
**Schnupp and Rosskothen-Kuhl**
The brains of human CI patients have become adapted to stimulation patterns in which TFS information is severely distorted. To really test the limits of temporal processing under cochlear implant stimulation, it is therefore helpful to turn to studies in experimental animals. Neonatally deafened, CI supplied rats exhibit excellent ITD sensitivity even at clinical stimulation rates if they had received timing cues from the onset of CI stimulation. This strongly suggests that the poor ITD sensitivity typically seen in human CI patients is not an inevitable consequence of biological limitations. CI rats show much greater ITD sensitivity to the timing of individual pulses than to the pulse-train envelope. Hence, the outcome with human CIs might be limited by existing stimulation strategies that encode only the envelope ITD. The early-deafened auditory pathway of CI rats is intrinsically exquisitely sensitive to both pulse timing ITDs and ILDs. Distorted fine structure cues provided by existing CIs are potentially highly misleading and could disrupt binaural hearing completely unless the pathway becomes desensitized to ITD. *A sensory diet that is enriched with appropriate pulse timing cues should lead to improvements in temporal processing in CI patients*.

The final bullet point in each box, shown in italics, contains suggestions for improving temporal-pitch and/or ITD processing.

Our first question concerned the neural basis for limitations in the processing of temporal cues to spatial location and to pitch. There was broad agreement that the limitation does not occur at the level of the AN, which conveys timing information even more precisely with electric than with acoustic stimulation. Several contributors note that the fidelity of AN phase-locking above the perceptual “upper limit” for ITD and for temporal pitch perception is observed both from invasive recordings from animals and from ECAP measures from human listeners. However, the abnormally synchronous response might impair phase-locking in brainstem neurons, perhaps by more-effectively engaging inhibitory mechanisms in the auditory brainstem (Delgutte & Chung; Vollmer & Ohl; Kral & Tillein). Regarding limitations occurring central to the AN, it was noted that many studies have reported a physiological “upper limit” in the phase-locked response to sustained monaural and binaural stimulation at the level of the IC and that is not present in the AN response. This limit contrasts to the very fine sensitivity of rat IC neurons to ITDs between pairs of isolated pulses (Buck et al., 2021), a difference that was attributed by Schnupp and Rosskothen-Kuhl to coarse sampling of ITDs in previous studies and by Delgutte and Chung to the use of single pairs of biphasic pulses compared to sustained pulse trains. Whatever the relative importance of these two factors, the relationship of a physiological upper limit to that observed perceptually requires parallel physiological and psychophysical measures in the same species. More generally, it seems clear that no single approach will provide the definitive answer. For example, human experiments are constrained in their ability to measure neural mechanisms in detail, whereas physiological recordings from animals do not reveal what the animal would actually hear. It is also possible that the upper limits differ between species, and so converging evidence from multiple animal models is likely to prove important.

Two sets of contributors (Carlyon & Deeks; Vollmer & Ohl) pointed out that the limitations in temporal processing are not exclusive to electrical stimulation; when analogous stimuli such as bandpass-filtered pulse trains are presented to NH listeners, the limitations are broadly similar to those of the best-performing CI listeners. In both cases, sensitivity at low rates is considerably worse than for low-frequency resolved harmonics presented acoustically to NH listeners. Furthermore, whereas ITD discrimination for pure tones increases with increasing frequency from 200 to 500 Hz, thresholds for the same task using bandpass-filtered harmonic complexes or modulated high-frequency tones deteriorate with increasing pulse rate over the same range (Bernstein & Trahiotis, 2002; Majdak & Laback, 2009). Hence, the processing of ITD and temporal pitch cues may be limited whenever changes in the temporal pattern of stimulation occur in the absence of either the corresponding place-of-excitation cues, the changes in the spectro-temporal pattern of stimulation that occurs with low-numbered resolved harmonics in NH (Carlyon et al., 2012; Cedolin & Delgutte, 2005, 2010; Larsen et al., 2008), or selective stimulation of the apex of the cochlea (Middlebrooks & Snyder, 2010).

There was, predictably, less agreement on the role of experience on the limits of temporal processing. Goldsworthy and others noted the substantial effects of training on rate-discrimination thresholds, whereas Carlyon & Deeks called for stronger evidence—such as from transfer of learning from a rate-discrimination to an ITD task—that these improvements reflected a genuine improvement in neural temporal processing. Several contributors pointed to the effect of early auditory experience on ITD processing in humans (Ehlers et al., 2017) and animals (Delgutte & Chung; Vollmer & Ohl; Schnupp & Rosskothen-Kuhl), but there was disagreement on the extent and basis of that effect in the animal literature. Furthermore, contributors disagreed about the effect of auditory experience and training in adulthood, both for ITD and for temporal pitch perception. Evidence that human CI listeners’ poor sensitivity to fine timing cues is not entirely due to auditory deprivation in childhood comes from observations that performance differs between electrodes within the same ear, and that ITD and pitch processing is worse than in NH even for patients deafened in adulthood. Fortunately, this disagreement led to several interesting suggestions for experiments that might resolve this issue, some of which fell within the remit of our question “what would change your mind?” These included a comparison of ITD sensitivity in unstimulated adult-deafened animals and those experienced with stimulation lacking ITD cues (Delgutte & Chung; Schnupp & Rosskothen-Kuhl), electrophysiological correlates of training effects in adulthood (Carlyon & Deeks), and measures of temporal pitch perception in very recently deafened individuals (Goldsworthy).

The debate also generated ideas for improving pitch and/or ITD processing for CI listeners, which are summarized in italics for each contributor in Table 2 and in some cases were proposed independently by different contributors. The possible deleterious effects of the abnormally high within- and across-neuron synchrony in the AN to electric pulse trains led several to suggest either jittering of the temporal pattern of stimulation and/or reducing the width of the excitation pattern (Carlyon & Deeks; Goldsworthy; Delgutte & Chung). Evidence for an apical pathway, selective for fine temporal processing (Middlebrooks & Snyder, 2009) led to the suggestion of producing selective apical stimulation, using either modifications of existing technology or new methods such as optogenetic or penetrating-nerve stimulation. Another potential route to improvement was the development of new speech-processing strategies. Both Goldsworthy and Carlyon & Deeks proposed strategies that presented the same TFS information to multiple electrodes, so as to provide a clear and consistent temporal pitch cue across a range of AN fibers. However, perhaps the most coherent call was for the development of strategies and/or hardware that preserve interaural timing cues. One such strategy (Peak Detection Timing, “PDT”: van Hoesel & Tyler, 2003) already exists experimentally, but has not been adopted as a clinical strategy and has produced mixed results when tested with experienced adult CI users. The arguments proposed here suggest that greater success might arise from either modified strategies that present consistent temporal patterns to adjacent electrodes or by providing such strategies at the time the CIs are first activated, thereby avoiding the maladaptive plasticity that may occur in patients who are used to conventional strategies in which uninformative and potentially misleading ITD information is presented (Schnupp & Rosskothen-Kuhl).
